# In Vivo Genome Editing in Type I and II Methanotrophs Using a CRISPR/Cas9 System

**DOI:** 10.1021/acssynbio.2c00554

**Published:** 2023-01-23

**Authors:** Bashir L. Rumah, Benedict H. Claxton Stevens, Jake E. Yeboah, Christopher E. Stead, Emily L. Harding, Nigel P. Minton, Ying Zhang

**Affiliations:** BBSRC/EPSRC Synthetic Biology Research Centre (SBRC), School of Life Sciences, Biodiscovery Institute, University of Nottingham, University Park, Nottingham NG7 2RD, U.K.

**Keywords:** methanotrophs, CRISPR, genome editing, methane monooxygenase, gene deletion, gene insertion, DNA ligase, promoter library, homology-directed repair

## Abstract

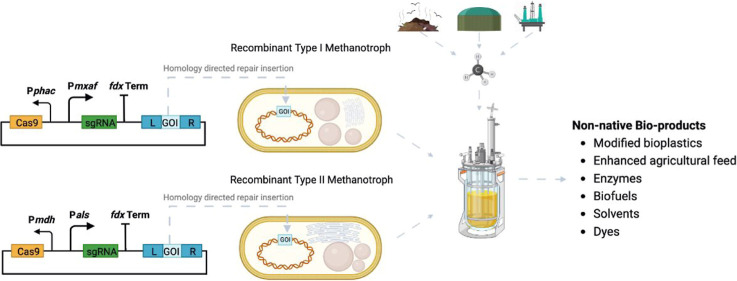

Methanotrophic bacteria are Gram-negative, aerobic organisms that use methane as their sole source of carbon and energy. In this study, we constructed and exemplified a CRISPR/Cas9 genome editing system and used it to successfully make gene deletions and insertions in the type I methanotroph *Methylococcus capsulatus* Bath and the type II methanotroph *Methylocystis parvus* OBBP. High frequencies of gene deletions and insertions were achieved in combination with homology-directed repair. In *M. parvus* OBBP, we also investigated the impact of several parameters on the CRISPR/Cas9 genome editing, where the *ligD* gene was targeted with various PAM sequences and guide RNA spacer sequences, homology arms of variable length, differences in the duration of mating during conjugation, and exploiting promoters of different strengths to control the expression of cas9 and sgRNA. Although not the first attempt to develop a CRISPR/Cas system in methanotrophs, this work demonstrated for the first time an efficient CRISPR/Cas9 system generating scarless clean gene deletions and insertions in methanotroph genomes.

## Introduction

Methane (CH_4_) ranks as the second most abundant anthropogenic greenhouse gas (GHG) next to carbon dioxide.^1^ It can serve as the sole source of carbon and energy source for a group of microorganisms called methanotrophs, which are either aerobic bacteria or anaerobic archaea.^2,3^ As methanotrophs represent the primary biological sink of methane in the atmosphere and soil, they are extremely important in helping to control the levels of methane in the environment^4^ and therefore play an important role in global climate change. In recent times, methanotrophs are also utilized in industrial biotechnology, most notably in the manufacture of animal feed in the form of single cell protein.^5^

Methanotrophs are ubiquitous and can be found growing in most places where methane is emitted, such as landfill sites, lake sediments, wetlands, and marine environments.^6−9^ The distinguishing characteristic that enables methanotrophs to oxidize CH_4_ and survive in different environments is their exclusive possession of a broad-spectrum methane monooxygenase (MMO) enzyme.^10^ This enzyme is present in two forms, each found in different parts of the cell. The particulate form (pMMO) is membrane-associated, while the soluble form (sMMO) is found in the cytoplasm.^11^ Based on the morphology of their internal structures, particularly the intracytoplasmic membrane (ICM) where pMMO is found, and other characteristics such as the method of carbon assimilation, methanotrophs are divided into type I and type II. The ICM of type I organisms resembles disc-shaped vesicles and can be present all over the cell, whereas in type II methanotrophs, the ICM appears as paired membranes assembled at the cell periphery usually running around the whole cell. Type I strains utilize the ribulose monophosphate pathway for carbon assimilation as opposed to the serine cycle of type II methanotrophs.^2^

Some genetic tools have been developed for both type I and II methanotrophs. However, few are available for rational genome editing. Examples include marker-exchange mutagenesis,^12^ the *cre-lox* system,^13^ and the *sacB*-based deletion system.^14^ Marker-exchange mutagenesis has been successfully demonstrated in *Methylocella silvestris*(12) and reported in *Methylosinus trichosporium*(15) for gene deletion (*mmoX*). This method, however, has a number of disadvantages, including (1) a relatively high risk of introducing unwanted insertions or deletions of genetic elements due to the large genetic changes that are made^16^ and (2) a low frequency of gene replacement;^12^ and (3) once inserted, markers cannot be reused. The *cre-lox* system used in *M. silvestris* lacks consistency in terms of the rate at which double-cross over events are achieved (ranging from 5 to 50%)^13,16^ and leaves a recombinase recognition site scar,^17^ which can potentially affect phenotype and can be responsible for recombination events where multiple genes are deleted. The *sacB*-based deletion system, as with the *cre-lox* system, makes marker-less genomic edits^18^ and is an easily applicable system designed for use in a wide range of organisms. As with all counter-selections systems, however, there is a high rate of false positives due to spontaneous sucrose-resistant colonies. Moreover, as the method requires the initial isolation of single cross-over integrants, followed by the subsequent isolation of double cross-over mutants, the two-step process is time-consuming when manipulating slow growing methanotrophs.

More recently, genome editing based on Clustered Regularly Interspaced Short Palindromic Repeats (CRISPR) has been developed as a quick and easy-to-design tool for a wide variety of living organisms.^19−21^ Its deployment in methanotrophs would potentially increase mutagenesis efficiency, leave no markers or scars, and decrease the time needed to generate the required edits, an important consideration in slow growing methanotrophs. Genome editing is achieved in CRISPR/Cas9 systems when DNA strand breaks induced by Cas proteins are repaired using repair templates (homology-directed repair—HDR) or using error-prone template-free cellular machinery (non-homologous end joining—NHEJ).^22^ Although genome editing using CRISPR has been demonstrated once in *Methylococcus capsulatus* Bath, the CRISPR system used was a Cas9^D10A^ nickase, the efficiency achieved was extremely low (2%), and no clean deletion of the targeted gene was demonstrated.^23^

Here, we demonstrate the deployment of a highly effective, *Streptococcus pyogenes*-based CRISPR/Cas9 system that generates scarless gene deletions and/or insertions in both a type I (*M. capsulatus* Bath) and type II (*M. parvus* OBBP) methanotroph. Two methods of gene insertion were developed and exemplified by genomic insertion of the eYFP reporter gene into the *ligD* of *M. parvus* OBBP, and one method involving eYFP reporter gene insertion (gene replacement) in MCA_0145 gene of *M. capsulatus* Bath. This work also provides a Tn*5* transposon-based strategy for identifying the non-essential genes to be targeted when first implementing genome editing tools, such as CRISPR/Cas9 in a microbe. The importance of identifying essential genes, though often overlooked, can be a deciding factor in the successful implementation of any new genome editing system as demonstrated in this study.

## Materials and Methods

### Bacterial Strains and Growth Conditions

Details of all bacterial strains used in this study are in Table SI. *Escherichia coli* XL-1 blue was used for routine cloning, *E. coli* DH5α was used for HiFi assembly, and *E. coli* S17-1 λ*pir* was used for bi-parental conjugation. Unless when making chemically competent cells, *E. coli* strains were grown at 37 °C in Lysogeny broth (LB) media supplemented with kanamycin 50 μg/mL, while shaking at 200 rpm. Methanotrophs were grown in nitrate mineral salt (NMS) media supplemented with 10 μM CuSO_4_·7H_2_O.^24^ Shaking was at 200 rpm for liquid cultures. *M. parvus* OBBP was incubated at 30 °C, while *M. capsulatus* Bath was incubated at 37 °C or 45 °C both with a 1:5 CH_4_/air mixture when grown in serum bottles. When methanotrophs were grown with solid media on plates, CH_4_ was gassed into anaerobic jars. During conjugation, kanamycin was used at 15 μg/mL for *M. capsulatus* Bath and 50 μg/mL for *M. parvus* OBBP. Nalidixic acid at 25 μg/mL was used for counterselection against *E. coli* S17-1 λ*pir* during conjugation.

### Promoter Strength Assays

The promoters in Table S2 were tested for activity in *E. coli* S17-1 λ*pir*, *M. parvus* OBBP, and *M. capsulatus* Bath. Most of the promoters were derived by cloning DNA regions upstream of the start codon of genes from *M. parvus* OBBP and *M. capsulatus* Bath. DNA regions approximately 300 bp from the start codon of selected genes were analyzed with three promoter prediction algorithms (Promoter Prediction by Neural Network, BPROM and PePPER)^25^ and the DNA sequences predicted to have promoters by at least two of the software packages were sent to Twist Bioscience for synthesis. After synthesis, each promoter was cloned upstream of a reporter gene encoding enhanced yellow fluorescent protein (eYFP) and transformed into *E. coli* S17-1 λ*pir*. Details of the cloning and plasmid design can be found in Supporting Information.

The various plasmids made were conjugated into *M. parvus* OBBP and *M. capsulatus* Bath as described below; the level of eYFP expression was estimated using fluorescence assays. Actively growing cultures were diluted to approximately OD_600_ 0.5. Quadruplicates of each were pipetted (100 μL) into Black Greiner 96 Well Flat Bottom (Chimney well) plates. Culture harbouring the control plasmid (plasmid with a promoter-less eYFP gene) was pipetted in wells (100 μL) in 12 replicates. The media was also pipetted (100 μL) into 12 different wells. Fluorescence was measured with a Tecan M1000 using the following parameters: excitation wavelength—495 nm; emission wavelength—530 nm; optical density wavelength—600 nm; mode—read from the bottom; gain—70; shaking frequency—408 rpm; kinetic cycles—5. Promoter activity was measured as fluorescence/OD, normalizing for fluorescence from media and the promoter-less eYFP reporter gene.

### Plasmid Design and Construction

Oligonucleotide primers used for cloning and sgRNA were synthesized by Merck UK and Eurofins in Germany and are listed in Table S3. Detailed plasmid construction is described in Supporting Information, and the plasmids used in this study are listed in Table S4. Sanger sequencing was carried out by Source Bioscience Nottingham.

### Conjugation of Methanotrophs and Mutant Screening

Conjugation was carried out based on modifications of the method used by Martin and Murrell.^26^*E. coli* S17-1 λ*pir* harboring the plasmid intended for conjugation was grown overnight in LB media containing kanamycin (50 μg/mL). The absorbance (OD_600_) of the grown overnight culture was measured, and the volume required to give 1 mL of *E. coli* S17-1 λ*pir* at OD_600_ of 1 was calculated and pipetted into an Eppendorf tube. For example, 0.4 mL of OD_600_ 2.5 gave 1 mL of OD_600_ 1. The *E. coli* S17-1 λ*pir* culture was then washed three times with NMS media to remove the antibiotics by centrifuging at 8000 rpm for 3 min. After the third wash, a calculated volume of methanotroph culture was mixed to have 1:1 donor/recipient ratio by OD. The mixture of *E. coli* S17-1 λ*pir* and methanotroph was spun at 8000 rpm for 3 min and resuspended in 50 μL of NMS, which was spotted on a dry plate of NMS Bacto Agar containing 0.5% yeast extract. The spot was allowed to dry, and the plates were placed in air-tight anaerobic jars. The jars were sparged for 3–5 s with CH_4_ and incubated at 30 °C for *M. parvus* OBBP and 37 °C for *M. capsulatus* Bath typically for 48 h unless otherwise stated. After 48 h of mating, the spot was scraped with a sterile loop and resuspended in 1 mL of NMS. The resuspension was diluted to 10^–7^ in NMS media. In triplicate, 10 μL of each dilution including neat was plated in a sector of LB agar and NMS Bacto agar plate divided into eight sectors with the required antibiotics. NMS Bacto agar plates were incubated for 2 weeks while LB agar plates were incubated overnight. After incubation, colonies that appeared on plates were counted. Conjugation efficiency (CE) was calculated as the number of transconjugants per donor cell. In other words, the number of methanotrophs that grew in a particular dilution in NMS Bacto agar plates with antibiotics per number of *E. coli* S17-1 λ*pir* that grew in the same dilution on LB media plate with antibiotics.^27^

After conjugating CRISPR/Cas9 plasmids into methanotrophs, to identify mutants generated by our CRISPR/Cas9 system, transconjugant colonies growing on NMS antibiotic plates were picked, patch-plated on NMS antibiotic plates, and dipped into a 25 μL PCR mix according to NEB UK Q5 high-fidelity polymerase instructions, using specific flanking primers (Table S3). In all cases, mutations were further confirmed with Sanger sequencing.

### Transposon Mutagenesis

To determine non-essential genes, transposon mutagenesis was used to generate mutant cells with transposon insertion in non-essential genes and gene regions. This was carried out by conjugation of *M. parvus* BRCS2 and *M. capsulatus* Bath with pMTL90531_Tn5 plasmid using *E. coli* S17-1 λ*pir*. The location of transposon insertion and subsequently designated non-essential gene was determined by inverse PCR and Sanger sequencing. Cloning pMTL90531_Tn5, conjugation of the plasmid, and inverse PCR are described in detail in Supporting Information.

### Plasmid Curing

During screening of colonies for mutants, each colony was patch-plated on NMS antibiotic plates before screening with PCR. After confirming genome editing in colonies using PCR, the patch plates were revisited and re-streaked on antibiotic-free NMS plates. After growth, a colony is transferred to NMS liquid media and allowed to grow to exponential phase. The culture is then diluted up to 10^–6^. Dilutions are then spotted on NMS plates in replicates. After growth on NMS plates, 25 colonies are scooped and dipped in PCR tubes containing 50 μL of NMS media. From this mixture, 5 μL is spotted on both NMS and NMS antibiotic plates. The presence of growth on NMS plates and absence of growth on NMS antibiotic plates suggest that the plasmid has been cured. The colony confirmed with plasmid cured cells is then grown in liquid NMS media and cryo-stocked. Example plates are shown in Figure S2.

## Results and Discussion

### Promoter Strength Assays

**Figure 1:**
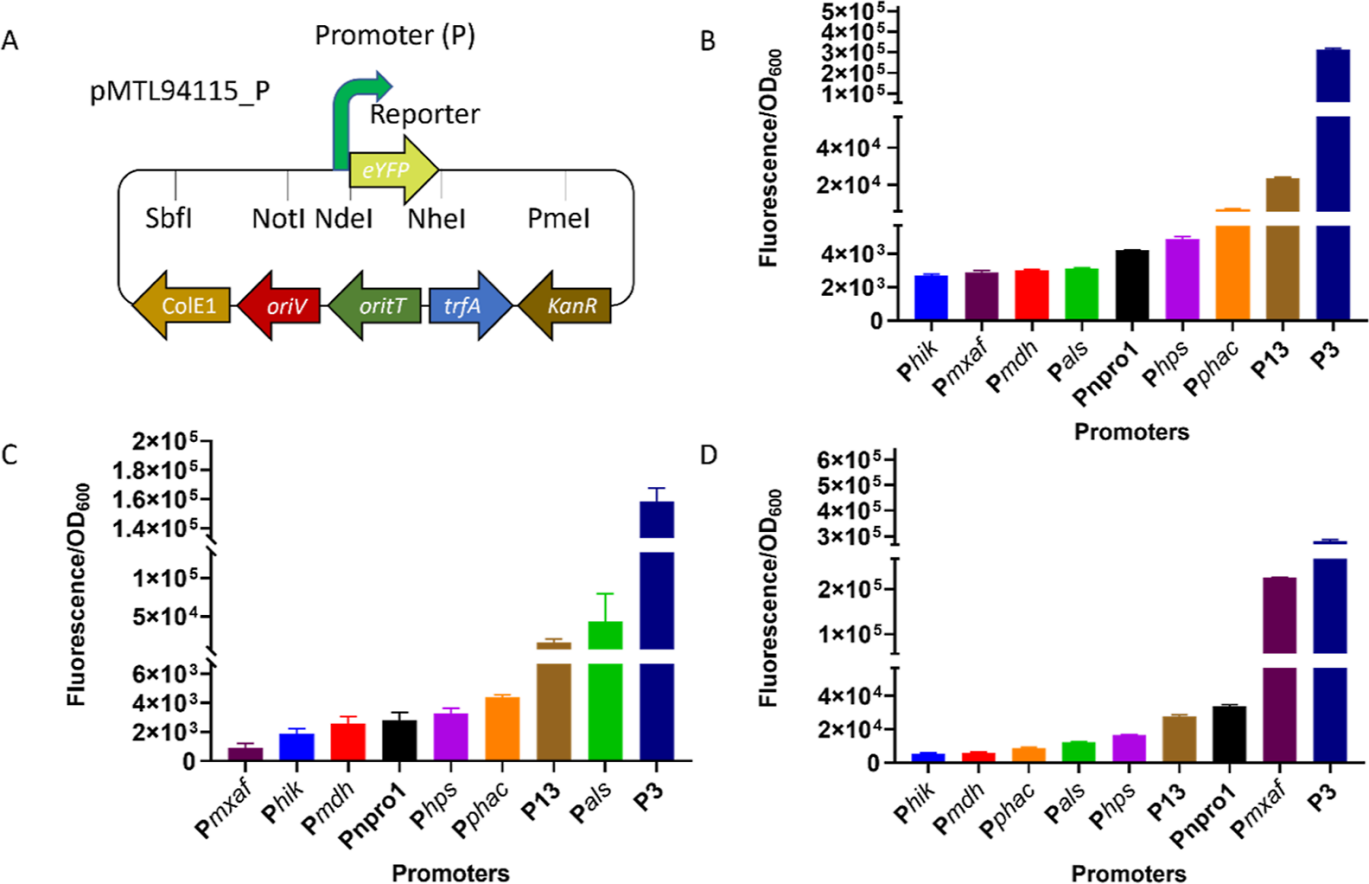
Fluorescence assay of promoters. (A) pMTL94115_P plasmid used for promoter assay. Fluorescence assay of promoters in (B) *E. coli* S17-1 λ*pir*; (C) *M. parvus* OBBP; (D) *M. capsulatus* Bath. All promoters are expressed constitutively using eYFP as the reporter. Fluorescence values for promoterless eYFP was used to normalize all promoter-eYFP data.

Although previous studies have evaluated the performance of the *mxaF* promoter,^23,28^ the focus here was on testing uncharacterized promoters in type I (*M. capsulatus* Bath) and type II (*M. parvus* OBBP) methanotrophs. These comprised synthetic and constitutive promoters native to organisms listed in Table S2. Their activity was tested in *E. coli* S17-1 λ*pir*, *M. parvus* OBBP, and *M. capsulatus* Bath using a reporter gene encoding eYFP. pMTL94115 modular plasmid which has eYFP inserted in the multiple cloning site was used as the backbone to give pMTL94115_P where P stands for the specific promoter used as shown in Figure 1A. Relatively speaking, the promoters P_*als*_ and P3 led to the moderate and high expression, respectively, of eYFP in *M. parvus* OBBP. Only a low level of eYFP expression occurred from the remaining promoters. In *M. capsulatus* Bath, the use of P_*hps*_ and P_*mxaf*_ led to moderate expression of eYFP compared to the high level of expression observed from P3. In *E. coli* S17-1 λ*pir*, the P3 promoter also led to the highest level of eYFP expression (Figure 1B–D). The relative strength of the promoters in the three hosts was subsequently taken into account in selecting the promoter to be used to express *cas9* and sgRNA in the developed CRISPR-based gene editing system.

It was important to assess the expression levels of promoters in *E. coli* S17-1 λ*pir* to avoid overexpression of *cas9* during cloning, as this could lead to reduced transformation efficiency as reported in *E. coli* ER1821.^29^ Hence, promoters that weakly express *cas9* and express guide RNAs at relatively stronger levels in methanotrophs were selected for use, and those such as P3, shown to direct very high level of expression in both *E. coli* and methanotrophs, were avoided. The weakest promoters were also not selected for use to avoid expression of *cas9* below levels required for efficient double strand break. Promoter expression levels proved to be different for the two methanotroph species under investigation. The selected promoters were expressed constitutively, which contrasts with many other systems that place *cas9* under inducible control.^30−32^ However, our study clearly demonstrates that constitutive expression of *cas9* can be just as effective for successful genome editing so long as the level of *Cas9* expression is low in the cloning host to avoid mutations and DNA rearrangement of the plasmid during cloning. Nonetheless, inducible promoter-controlled *cas9* is advantageous in organisms with relatively low DNA transfer efficiencies, weak recombinases, and organisms that are susceptible to Cas9 toxicity.^33^ For example, in a study involving *Clostridium acetobutylicum*, no colonies were observed after transformation when *cas9* was under the transcriptional regulation of constitutive promoters, whereas efficient editing was observed when *cas9* was regulated with inducible promoters.^30^ This supports the importance of CRISPR system designs that incorporate inducible promoters such as those seen in the methanotroph study referred to earlier.^23^ Further experiments in this study were designed to investigate the toxicity of Cas9 in methanotrophs and CE of *Cas9* plasmids. It was decided to use promoter P_*phaC*_ to express *cas9* gene and promoter P_*mxaF*_ to express guide RNA in type I methanotroph *M. capsulatus* Bath. In type II methanotroph *M. parvus* OBBP, promoter P_*mdh*_ was used to express *cas9* gene and promoter P_*als*_ to express sgRNA.

### Establishment of a CRISPR/Cas9 Genome Editing Plasmid System in *M. parvus* OBBP

**Figure 2:**
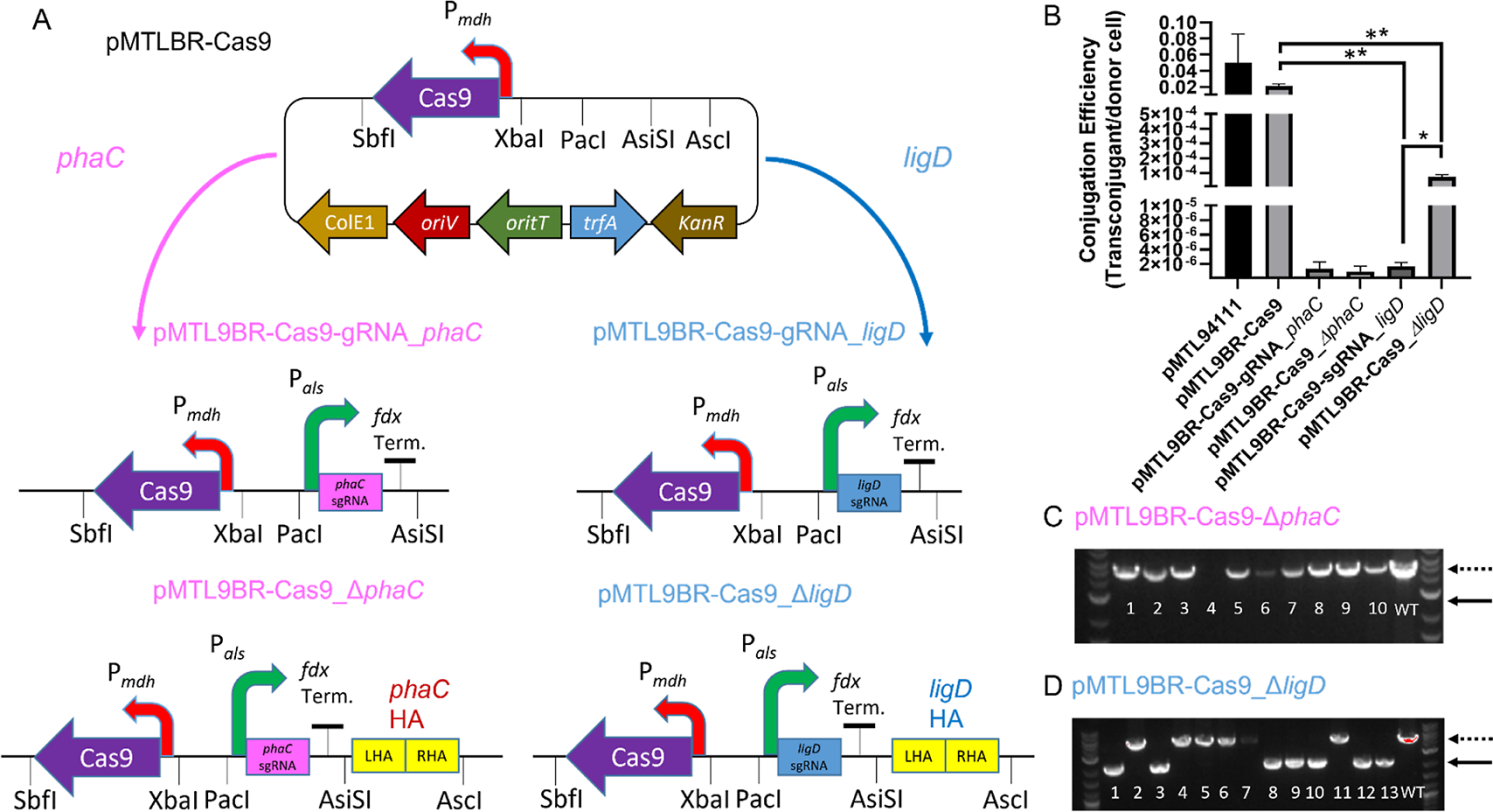
Plasmids, CE, and genome editing screens of *M. parvus* OBBP. (A) Plasmid pMTL9BR2-Cas9 carrying *S. pyogenescas9* gene cloned into pMTL94111 backbone and subsequent plasmids designed from pMTL9BR2-Cas9. (B) CE graph showing significant difference between pMTL9BR2-Cas9 when compared to pMTL9BR2-Cas9-gRNA_*phaC* or pMTL9BR2-Cas9_Δ*ligD* (*P* = 0.0088). pMTL9BR2-Cas9-gRNA_*ligD* CE was significantly lower than pMTL9BR2-Cas9_Δ*ligD* (*P* = 0.02). There was no significant difference between pMTL94111 and pMTL9BR2-Cas9 (*P* = 0.3681). Unpaired *t*-test used, *n* = 2, error bars represent the standard error of the mean. (C) Bands of PCR screens of unsuccessful *phaC* gene deletion. *phaC* is a 2070 bp gene, and edited colonies were expected to have bands approximately 2070 bp smaller than the control (WT), which is in the last lane. (D) Bands of PCR screen of successful *ligD* gene deletion. *ligD* is 2448 bp in size, and edited colonies were expected to have bands approximately 2448 bp smaller than the control (*M. parvus* OBBP WT gDNA), which is in the last lane. The numbers of colonies screened were represented by numbers. NEB 1 kb Plus DNA ladder was used. Dotted lines represent the size of WT PCR amplicon, while solid lines represent mutants.

CRISPR/Cas9 plasmid systems were designed in stages that will enable the functional study of various components such as *cas9*, guide RNAs, and repair templates in the form of homology arms. To investigate the specific effect of Cas9, for instance its toxicity in *M. parvus* OBBP, pMTL9BR2-Cas9 was constructed. In this plasmid, *cas9* was under the transcriptional regulation of a methanol dehydrogenase promoter P_*mdh*_. Second, the additional effect of guide RNA was investigated by cloning into pMTL9BR2-Cas9 a guide RNA with spacer targeting either *phaC* or *ligD* genes, resulting in plasmids pMTL9BR2-Cas9-sgRNA-*phac* and pMTL9BR2-Cas9-sgRNA-*ligD,* respectively. In both cases, the sgRNAs were driven by acetolactate synthase promoter P_*als*_. Finally, complete CRISPR/Cas9 plasmids pMTL9BR2-Cas9_Δ*phaC* and pMTL9BR2-Cas9_Δ*ligD,* which consist of *cas9*, sgRNA, and homology arms, were designed for Cas9-induced double strand break with the targeting effect of sgRNA and the repair function of homology arms (Figure 2A). The combined action of these three components was expected to result in genome editing. Details of cloning can be found in Supporting Information.

### Initial *M. parvus* OBBP Deletion Target, *phaC*

The first *M. parvus* OBBP gene to be targeted, *phaC*, encoded poly(*R*)hydroxybutyrate (PHB) synthase. Its selection was based on the fact that it has been deleted in other PHB-producing species such as *Cupriavidus necator,*(34) and it represents an important biotechnological target since its deletion would potentially allow carbon flux to be re-directed from PHB into alternative biosynthetic products. Using the promoters selected above and the modular plasmid pMTL94111, an appropriate CRISPR/Cas9 plasmid targeting *phaC* was constructed expressing *cas9* and sgRNA against *phaC* from the P_*mdh*_ and P_*als*_ promoters, respectively, together with a repair template comprising 1000 bp left and right homology arms flanking the intended 2070 bp *phaC* gene (pMTL9BR2-Cas9_Δ*phaC*) (Figure 2A). Control plasmids were also constructed lacking the repair template (pMTL9BR2-Cas9-sgRNA-*phaC*) or repair template and sgRNA (pMTL9BR2-Cas9). All three plasmids, together with the pMTL94111 progenitor, were conjugated into *M. parvus* OBBP and their CE estimated from the number of colonies obtained (Figure 2B). Plasmid pMTL9BR2-Cas9 transformed at the same high frequency as its progenitor, pMTL94111 (Figure 2B), suggesting that expression of Cas9 in the absence of a sgRNA is not toxic in *M. parvus* OBBP. This is in contrast to other microorganisms where expression of Cas9 can reduce transformation frequencies by two orders of magnitude.^29^ Although the non-toxic effect observed could indicate that Cas9 was not being produced, nevertheless, the plasmid carrying a combination of *cas9* and sgRNA targeting *phaC* (pMTL9BR2-Cas9-sgRNA-*phaC*) exhibited a significant reduction in CE (Figure 2B), consistent with the production of a functional Cas9-sgRNA complex and a reduction in cell numbers through double strand cleavage of the chromosome.^30^ An equivalent low frequency was seen with pMTL9BR2-Cas9_Δ*phaC,* suggesting that the addition of homology arms to the pMTL9BR2-Cas9-sgRNA-*phaC* did not increase the chances of transconjugants surviving. Colonies were screened to establish whether *phaC* deletion mutants had been generated using genome-specific primers flanking the repair template homology arms. No mutants were detected in three independent experimental attempts to delete *phaC* (Figure 2C). Failure to isolate *phaC* deletion mutants was suspected to be due to low homologous recombination (HR) efficiency in *M. parvus* OBBP. Past studies have reported that HR efficiency was increased by inhibiting or knocking out NHEJ genes such as the ATP-dependent DNA ligase *ligD*.^35,36^ A recent study reported 22% of bacteria carry this pathway.^37^ In common with 22% of bacteria, *M. parvus* OBBP appears to carry this pathway.^27^ Attempts were, therefore, made to delete *ligD*.

### *ligD* Gene Deletion in *M. parvus* OBBP

**Figure 3:**
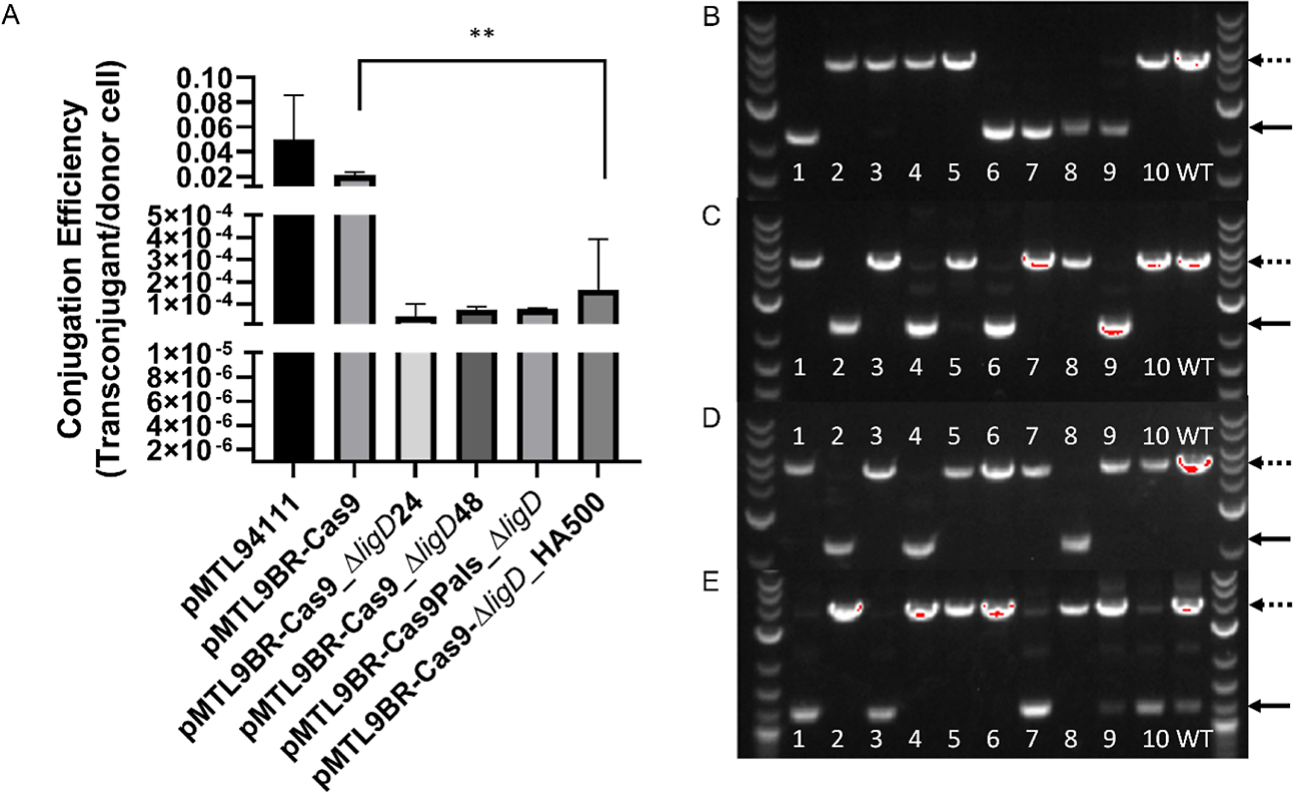
Δ*ligD* gene deletion under different conditions (A) CE of additional *ligD* deletions in *M. parvus* OBBP. (B) *M. parvus* OBBP *ligD* gene deletion using pMTL9BR2-Cas9_Δ*ligD*24 plasmid conjugated with *E. coli* (24 h mating time). (C) *M. parvus* OBBP *ligD* gene deletion using pMTL9BR2-Cas9_Δ*ligD*48 plasmid conjugated with *E. coli* (48 h mating time). (D) *M. parvus* OBBP *ligD* gene deletion using pMTL9BR2-Cas9Pals_Δ*ligD* plasmid with a relatively stronger Cas9 promoter (P_*als*_) compared to sgRNA promoter (P_*mdh*_) conjugated with *E. coli*. (E) *M. parvus* OBBP *ligD* gene deletion using pMTL9BR2-Cas9-Δ*ligD*_HA500 plasmid with 500 bp HA conjugated with *E. coli*. NEB 1 kb Plus DNA ladder was used. Dotted lines represent the size of WT PCR amplicon, while solid lines represent mutants. The number of colonies screened were represented by numbers.

To target *ligD*, equivalent plasmids to those made for *phaC* were constructed (Figure 2A) and conjugated in *M. parvus* OBBP from an *E. coli* S17-1 λ*pir* donor. After 2 weeks of incubation at 30 °C, colonies were counted, and the CE was estimated. Similar to the observation made with the *phaC* specific constructs, the combined presence of Cas9 and sgRNA directed against *ligD* significantly reduced the transformation frequency of the plasmid concerned, pMTL9BR2-Cas9-sgRNA-*ligD*, compared to pMTL9BR2-Cas9. This observation further supports our contention that the designed system is able to produce a functional Cas9/sgRNA complex. However, in the case of targeting *ligD*, the presence of a repair template for *ligD* on plasmid pMTL9BR2-Cas9_Δ*ligD*, together with *cas9* and sgRNA targeting *ligD*, significantly increased the CE, suggesting that the addition of homology arms to the plasmid increased the chances of transconjugants surviving by enabling removal of the PAM target through allelic exchange (Figure 2B,C). Evidence that this had occurred was provided by PCR screening of the colonies and the demonstration of DNA bands on agarose gels of a size consistent with the intended deletion in the *ligD* gene (Figure 2D). Gel extraction of these DNA fragments and their analysis by Sanger sequencing confirm that they were *ligD* gene deletions. This represents the first reported example of wild type *S. pyogenes* CRISPR/Cas9-mediated scarless genome editing in methanotrophs. The editing efficiency was 58%. Having demonstrated that the system was working, the effect of various parameters was investigated with a view to optimizing the process. These included mating times during conjugation, sgRNA spacer target score, promoter strength, and the length of homology arms used in the repair template. A reduction in the size of the homology arms used from 1000 to 500 bp in plasmid pMTL9BR2-Cas9-Δ*ligD*_HA500 appeared to have little effect on editing frequencies (Figure 3E) and gave the highest CE (*P* = 0.089) among the CRISPR/Cas9 plasmids tested (Figure 3A). Similar, if not slightly better, editing efficiency was demonstrated when mating times were reduced from the routinely used 48 to 24 h (Figure 3B,C). This reduction could represent a significant saving in the time needed to generate mutants. This contrasts with studies in *Bacillus subtilis* where additional incubation of the transformation mixture led to an increase in editing efficiency from 16 to 80%.^38^ Genome editing was also successful when a promoter of moderate strength was used for *cas9* (P_*als*_) expression and a relatively weaker promoter for gRNA (P_*mdh*_) (Figure 3D). This provides more flexibility when cloning CRISPR/Cas9 plasmids for genome editing. The ability of *M. parvus* OBBP to withstand moderate expression of Cas9 further supports its tolerance to Cas9 toxicity unlike in other organisms such as *Clostridium* species.^29^ In these experiments, genome editing efficiency varied from 30 to 50%, as shown in Figure 3.

Further experiments to understand the effect of different sgRNAs on genome editing showed that six additional sgRNAs successfully mediated the deletion of *ligD* regardless of the on-target score, genomic DNA strand, PAM, and position of spacer sequence along *ligD* gene (Table 1). This allows for a high level of flexibility in selecting the sgRNA to be used. With the sgRNA AltKO_ligDSeed3, up to 90% genome editing efficiency was achieved. Overall, of the parameters investigated, changing the sgRNA gave the highest variation in genome editing efficiency (10–90%) and likely represents the most important starting point when trying to optimize genome editing efficiency.

**Table 1:** Effect of gRNA Spacer Sequence and PAM on Success of CRISPR/Cas9 Gene Deletion in *M. parvus* OBBP^a^

name	seed code	GC (%)	on target score	off target score	strand	PAM	dist. along gene/gene length in bp (%)	actual dist. from end (bp)	genome editing eff. (%)
*ligD* Seed	GGAAGCGGGCTGTCCAATCG	65	64.3	100	+ve	agg	1616/2488 (65)	872	58
AltKO_*ligD*Seed1	CGAGAGGATGGTCTTCCGTG	60	73.4	100	+ve	cgg	2183/2488 (88)	305	50
AltKO_*ligD*Seed2	ATGGTCGCGAATTTCCCCCG	60	71.8	100	+ve	cgg	438/2488 (18)	438	50
AltKO_*ligD*Seed3	CATCACCCATGCAAGCCGGG	65	68.1	100	–ve	tgg	827/2488 (33)	827	90
AltKO_*ligD*Seed4	GCGCCATATAAAGTTCGTCG	50	71	100	+ve	tgg	614/2488 (25)	614	80
AltKO_*ligD*Seed5	TTTCAGCTCGAAGAGCCAAT	45	64.2	100	+ve	cgg	1704/2488 (68)	784	60
AltKO_*ligD*Seed6	GATCAAGGGCGACTTTCGAG	55	66.5	100	–ve	agg	1211/2488 (49)	1211	60

a Percent knockouts achieved from 10 sampled colony PCR’s except *ligD*_Seed which was from 12.

### Other Gene Targets for CRISPR/Cas9 Genome Editing in *M. parvus* OBBP

**Figure 4:**
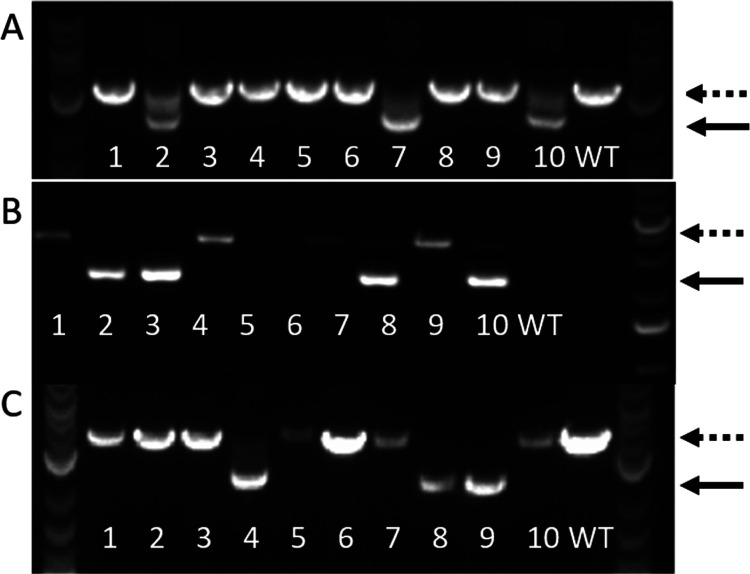
Deletion of additional genes in *M. parvus* OBBP. (A) *M. parvus* OBBP gene deletion using pMTL9BR2-Cas9_Δ*pntA* plasmid. (B) *M. parvus* OBBP gene deletion using pMTL9BR2-Cas9_Δ*bcsB* plasmid. (C) *M. parvus* OBBP gene deletion using pMTL9BR2-Cas9_ΔMPA_0518 plasmid. NEB 1 kb Plus DNA ladder was used. Dotted lines represent size of WT PCR amplicon, while solid lines represent mutants. Lanes 1–10 represent the 10 colonies screened.

Having generated a *ligD* mutant, additional genes were targeted, including *glg* (a gene involved in glycogen metabolism), *copD* (a copper tolerance gene), and *ligA* (an ADP-dependent DNA ligase). No evidence of gene deletion was obtained. In addition to low HR efficiency earlier suspected when *phaC* failed to be deleted, another potential reason to explain the failure to delete a particular target was that the gene was essential. Non-essential genes may be potentially identified through their inactivation using a transposon. Accordingly, a Tn*5*-based transposon plasmid pMTL90531_Tn5 was assembled and conjugated in *M. parvus* BRCS2 to create random transposon mutants (Figure S1). This particular methanotroph was chosen, because unlike *M. parvus* OBBP whose genome is only in draft form, the *M. parvus* BRCS2 genome is complete.^27,39^ The genomes of the two strains in any case share 99.99% homology. After screening a number of colonies with inverse PCR, 37 transposon insertion sites within *M. parvus* BRCS2 genome were identified, and the genes affected were identified by nucleotide sequencing. This led to the identification of three non-essential genes, which were identical in the DNA sequence to the type strain *M. parvus* OBBP, namely, *pntA* (an NAD(P) transhydrogenase subunit), MPA_0518 (a hypothetical protein), and *bcsB* (a cyclic di-GMP binding protein precursor). Equivalent CRISPR/Cas9 plasmids to those used to knock out *ligD* were constructed and shown to generate mutants in the three genes of *M. parvus* OBBP with 30–40% genome editing efficiency (Figure 4A–C). The colonies with edited genomes were re-streaked on kanamycin plates and subsequently grown in kanamycin-free media to start a plasmid curing process. Up to 100% plasmid curing rate was obtained based on 25 colonies for *bcsB* gene deletion. As with *ligD*, further experiments using eight additional sgRNA spacers were carried out to investigate gene deletion success in these new targets. The use of six of the eight alternative sgRNAs led to successful gene deletion (Table S5).

This finding emphasizes the need to choose gene targets for the exemplification of new genome editing systems that are proven to be non-essential as opposed to hypothesized to be based on indirect findings, for example, *phaC* is shown to be dispensable in other organisms such as *C. necator*.^34^ Hence, our strategy of using transposons to identify non-essential genes provided ideal targets to validate our genome editing system and would make a significant difference whenever such systems are being developed in new and understudied organisms. Once non-essential genes were identified, the deletion of 4 genes using 16 different sgRNAs was easily achieved.

### Effect of *M. parvus* OBBP ATP-Dependent DNA Ligase (*ligD*) on Genome Editing Efficiency

The successful knock-out of *ligD* may have been a consequence of the ablation of NHEJ, which compelled the cell to repair its genome via HDR. *ligD* inhibition has been shown to increase the efficiency of HR in rice^35^ and to increase genome editing efficiency in mammalian cells and mice.^36^ Therefore, it was hypothesized that deletion of *phaC* could succeed if the function of *ligD* was compromised, leading to an increased HR and genome editing efficiency. To test this, *phaC* gene deletion gene was pursued in a Δ*ligD*^*–*^ mutant strain. In a second experiment, *phaC* was targeted in the presence of the *ligD* inhibitor SCR7 added to the media during conjugation. No gene deletion was observed in either case. This outcome strongly suggests the essentiality of *phaC* and casts doubts to any significant role played by *ligD* with regard to HR and gene editing efficiency.

The effect on the editing efficiency of a gene that could be knocked out when performed in a Δ*ligD* background was further explored. Accordingly, the efficiency of gene editing of MPA_0518 in a wild type and Δ*ligD* background was compared. MPA_0518, a gene coding for a hypothetical protein, was already knocked out in this study. The deletion efficiency was higher in the wild type (61%) compared to the Δ*ligD* mutant strain (50%), suggesting that the *ligD* deletion does not offer genome editing advantages (Figure S4).

### Gene Insertion in *M. parvus* OBBP

**Figure 5:**
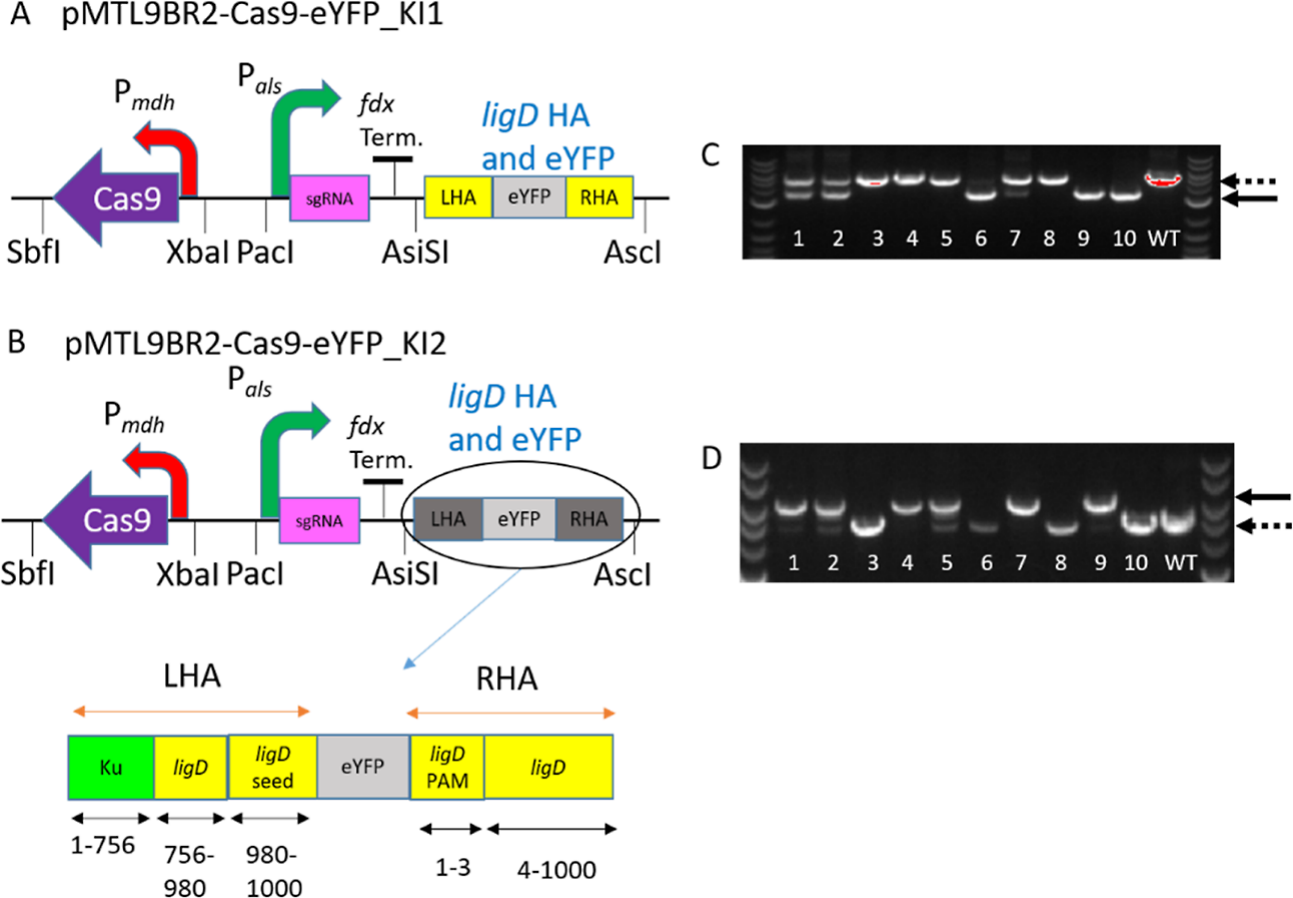
Gene insertion in *M. parvus* OBBP. (A) plasmid pMTL9BR2-Cas9-eYFP_KI1 used for inserting eYFP gene and simultaneous replacement of *ligD* gene. (B) Plasmid pMTL9BR2-Cas9-eYFP_KI2 used for insertion of eYFP gene without gene replacement. (C) eYFP and its P3 promoter are both 1091 bp long. Using pMTL9BR2-Cas9-eYFP_KI1 to Replace *ligD* with eYFP will give a band around 1357 bp smaller than the control which is *M. parvus* OBBP WT gDNA. (D) pMTL9BR2-Cas9-eYFP_KI2 colonies with successful gene insertions have bands approximately 1091 bp higher than the control, which is *M. parvus* OBBP WT gDNA. The same primer set was used for screening in (C,D). NEB 1 kb Plus DNA ladder was used. Dotted lines represent size of WT PCR amplicon, while solid lines represent mutants. Lanes 1–10 represent the 10 colonies screened.

After the successful deletion of multiple gene targets in *M. parvus* OBBP, CRISPR/Cas9 plasmids were designed to insert DNA cargo into *M. parvus* OBBP genome. A gene encoding eYFP under the transcriptional regulation of a strong promoter (P3) was chosen for the proof-of-concept experiments. Two approaches were investigated. In the first, gene replacement approach, the eYFP gene was introduced concomitantly with the deletion of *ligD* (Figure 5A), through its insertion between two 1000 bp homology arms flanking the *ligD* gene. This method was demonstrated in *Corynebacterium glutamicum,* where *ldhA* was replaced with a *rfp* cassette.^40^ In the second approach, the region between *ligD* sgRNA spacer and PAM was targeted for insertion. In this design, the left homology arm extended 1000 bp all the way to the end of the gRNA spacer, while the right homology arm started from the PAM extending 1000 bp in the 5′–3′ direction. The inserted eYFP gene was positioned between the two homology arms (Figure 5B). This is a slight modification of the method used in *Clostridioides difficile* where an anaerobic active green fluorescent protein was inserted between the nucleotide 101 and 102 downstream from a gene terminator.^41^ Both methods led to the successful insertion of the eYFP gene with 60% efficiency, as confirmed by PCR and Sanger sequencing of the amplified product.

### Gene Deletion in *M. capsulatus* Bath

**Figure 6:**
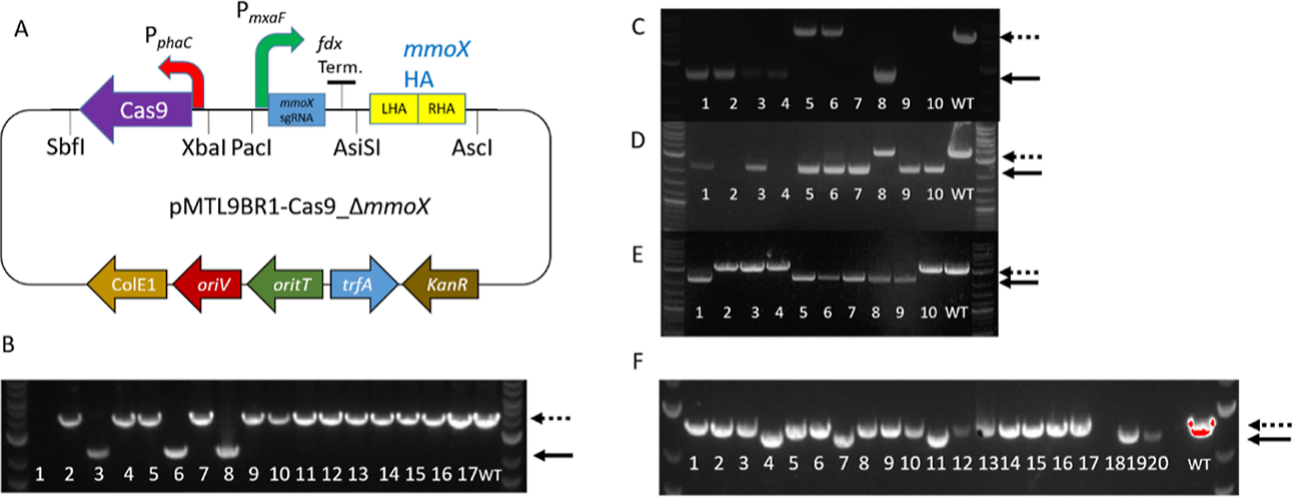
Genome editing in *M. capsulatus* Bath. (A) *M. capsulatus* Bath *mmoX* gene deletion plasmid pMTL9BR1-Cas9_Δ*mmoX*. (B) *M. capsulatus* Bath *mmoX* gene deletion PCR screen. (C) *M. capsulatus* Bath gene deletion using pMTL9BR1-Cas9_Δ*czcA* plasmid. (D) *M. capsulatus* Bath gene deletion using pMTL9BR1-Cas9_ΔMCA_0145 plasmid. (E) *M. capsulatus* Bath gene deletion using pMTL9BR1-Cas9_ΔMCA_2158 plasmid. (F) Gene insertion of eYFP with simultaneous gene deletion of MCA_0145 using plasmid pMTL9BR1-Cas9_ΔMceYFPKI1. NEB 1 kb Plus DNA ladder was used. Dotted lines represent the size of WT PCR amplicon, while solid lines represent mutants.

To test the versatility of the developed method to other methanotrophs, *M. capsulatus* Bath was targeted. The two targets selected were *mmoX* and *ligA*. The former was considered non-essential as it has been disrupted previously,^23^ while the latter was suggested to be involved in a possible NHEJ system referred to as alternative end-joining. Inactivation of *ligA* could potentially increase the efficiency of genome editing.^42^ Though *ligA* is an NAD-dependent DNA ligase, unlike *ligD* which is ATP-dependent and proven to increase genome editing efficiency when inhibited in mammalian cell lines and plants, as earlier reported.^35,36^ For *M. capsulatus* Bath the CRISPR/Cas9 plasmids constructed used the P_*phac*_ and P_*mxaf*_ promoters to control the expression of *cas9* and sgRNA, respectively (Supporting Information). The *mmoX*-specific CRISPR/Cas9 plasmid (pMTL9BR1-Cas9_Δ*mmoX*) produced gene deletions with 19% efficiency (Figure 6B). However, the use of the *ligA*-specific CRISPR/Cas9 plasmid did not result in the isolation of deletion mutants. This is likely due to the essentiality of *ligA*, as is the case in *B. subtilis*.^43^ To identify further non-essential gene targets, the transposon approach developed in *M. parvus* OBBP was implemented (Supporting Information). Among the non-essential genes identified in *M. capsulatus* Bath, three were targeted using our CRISPR/Cas9 system. These were genes encoding a porin family membrane protein (MCA_0145), a heavy metal efflux pump (*czcA*), and a transcriptional regulator from the *AraC* family (MCA_2158). Using appropriately constructed CRISPR/Cas9 vectors, deletion mutants of the three genes were isolated with an efficiency of 50, 70, and 60% for MCA_0145, *czcA,* and MCA_2158, respectively (Figure 6C–E). Plasmids of all successful knockouts in *M. capsulatus* Bath were cured using the same method for *M. parvus* OBBP with up to 84% plasmid curing rate based on 25 colonies for *mmoX* gene.

### Gene Insertion in *M. capsulatus* Bath

Having demonstrated that the CRISPR/Cas9 system was able to generate deletions, the ability of the system to mediate knock-in was tested essentially as exemplified in *M. parvus* OBBP using the eYFP gene as cargo via the gene replacement approach. In the initial experiments, *mmoX* was targeted, but very few colonies were obtained following conjugation with the requisite CRISPR/Cas9 insertion vector and none carried the desired eYFP insertion. Accordingly, the gene replacement target was changed to the MCA_0145 gene as its deletion efficiency was higher than *mmoX*. Initially, the number of colonies obtained after conjugation remained low (less than 3 colonies per plate) and no eYFP insertion mutants were isolated. To improve the number of colonies obtained after conjugation, a few modifications were made to the conjugation protocol including mating at 30 °C instead of 37 °C, doubling the number of mating and donor cells, increasing the concentration of CaCl_2_ in the media 10-fold and, finally, halving the antibiotic concentration used to 7.5 and 12.5 μg/mL of kanamycin and nalidixic acid, respectively. Of these modifications, only reducing the antibiotic concentration resulted in a significant increase in the number of colonies, which led to the successful gene insertion of the eYFP gene under the transcriptional control of the P3 promoter. A 25% gene insertion efficiency was obtained (Figure 6F).

So far, our study has demonstrated, for the first time, two methods of gene insertion in *M. parvus* OBBP and one method in *M. capsulatus* Bath. They pave the way for the rapid introduction of exogenous genetic pathways into the genome of methanotrophs, promoting a desirable chassis for biotechnological applications. The genome editing efficiency was also monitored and was usually around 30–50%, although up to 90% genome editing efficiency was attained in some instances. The non-edited colonies in genome editing experiments often referred to as escapers survive for several reasons.^33^ Among them are possible *cas9* gene mutation in plasmids found in escaper cells and/or cellular modification of sgRNA target site in CRISPR/Cas9 plasmids or DNA of such cells. We found that CRISPR/Cas9 genome editing in *M. parvus* OBBP was successful regardless of homology arm length, duration of mating in conjugation, and promoter expression levels as long as the promoter controlling *cas9* gene was not highly expressed in *E. coli*. However, the choice of sgRNA is crucial, as demonstrated in two experiments targeting the hypothetical protein MPA_0518 gene; two sgRNAs did not result in gene deletions suggesting that more than one sgRNA should be tested in parallel.

To provide more understanding around why this study led to successful wild type Cas9-initiated HDR-assisted scarless genome editing compared to a previous study,^23^ it is important to highlight differences between both studies. The first consideration is the methanotrophic bacteria used. In our study, type I and II methanotroph genomes were edited using wild type *S. pyogenescas9* and HDR was used for DNA repair after Cas9-induced double strand break. In the previous study,^23^ genome editing of only the type I *M. capsulatus* Bath was attempted. Wild type *S. pyogenescas9* did not lead to successful genome editing, and NHEJ was used instead of HDR when success was achieved using the mutated Cas9^D10A^ nickase to achieve genome editing via nonsense mutation.

With regards to plasmid design, different promoter sets were used for genome editing in type II *M. parvus* OBBP involved in our study. For type I *M. capsulatus* Bath plasmid design where there are more similarities in both studies, the first difference observed was the *cas9* promoter used. In our study, the *phaC* promoter from *C. necator* H16 was constitutively expressed. P_*phaC*_ is a relatively weak promoter in both *E. coli* that was used as a cloning and conjugative host, as well as in *M. capsulatus* Bath which possesses the genome target. In the previous study,^23^ the inducible tetracycline promoter (P_*tetA*_) was used. P_*tetA*_ can lead to strong transcription of *cas9* depending on the concentration of the inducer (anhydrotetracycline) added. A high level of *cas9* expressions can lead to plasmid DNA mutation in regions essential for the functioning of the CRISPR-Cas9 systems. Another difference is in the guide RNA spacer used. Whereas our selection of guide RNA spacers was guided by tools provided by Benchling and CRISPy, it is unclear what guided spacer selection in the previous study. As noted in our spacer investigation, guide RNA spacer may determine efficiency and ultimately success of genome editing. Additionally, the guide RNA terminators used in both studies were likely different. Finally, we used a one-plasmid system, whereas a two-plasmid system seemed to have been used in the previous study.^23^ One or a combination of the factors highlighted may have been crucial in efficient wild type Cas9-initiated HDR-assisted scarless genome editing.

The demands to extend advanced synthetic biology techniques to more exotic chassis are high, especially for the development of industrial biocatalysts in an array of non-model microbes. In this study, we developed a broad-host-range CRISPR/Cas9 system and demonstrated the efficient genetic manipulation of type I methanotroph *M. capsulatus* Bath as well as type II *M. parvus* OBBP. Furthermore, our challenges of identifying non-essential gene knockout targets highlight the dire need for basic research on bacterial molecular biology in this class of ubiquitous yet understudied organisms. We anticipate that novel molecular mechanisms underlying methanotroph biology will be probed and accelerated with the addition of CRISPR/Cas9 to the limited methanotroph genetic toolbox.

## Supporting Information Available

Plasmid design and cloning; alternative CRISPR knock-outs spacers; strains used in this study; promoters and origin; primers used for designing plasmids and screening gene deletions; plasmids used in this study; transposon mutagenesis plasmid; plasmid curing on NMS agar plates; knock-out success percentages of the 18 tested CRISPR knock out spacer; and gene knock out in *M. parvus* OBBP wild type strain and *M. parvus* OBBP Δ*ligD* strain (PDF)

## Notes

All data generated or analyzed during this study are included in this published article [and Supporting Information].
