# Supplementary material for: In Vivo Genome Editing in Type I and II Methanotrophs Using a CRISPR/Cas9 System
Source: ACS Synth Biol. 2023 Jan 23;12(2):544–54. doi: 10.1021/acssynbio.2c00554 (PMC9942187; doi:10.1021/acssynbio.2c00554)
Supplement: Supplementary file 1 — sb2c00554_si_002.pdf [file sb2c00554_si_002.pdf]

Supporting information for

In vivo genome editing in type I and II methanotrophs using a CRISPR/Cas9 system

Bashir L. Rumah, Benedict H. Claxton Stevens, Jake E. Yeboah, Christopher E. Stead, Emily L. Harding, Nigel P. Minton and Ying Zhang*

*Corresponding author. Email: Ying.Zhang@nottingham.ac.uk

Plasmid design and cloning

Plasmids used in this study can be traced back to the SBRC Nottingham modular plasmid pMTL94111 (https://plasmidvectors.com/) which has oriV, oriT, trfA, ColE1, kanamycin resistant cassette and a multiple cloning site. To make this plasmid, pMHA199 reported to work for methanotrophs, was first assembled using genes from three plasmids purchased from BIOMATIK – pBSK1, pBSK2 and pJET1.2 [1]. These plasmids were electroporated into *E. coli* DH5α and plated on LB ampicillin (100µg/mL). Colonies from all three plates were grown and miniprep was used to extract plasmids. Plasmids of pBSK1, pBSK2 and pJET1.2 were digested with NotI/BamHI, NotI/XhoI and BamHI/XhoI respectively. The digests were ligated to form pMHA199. PCR of pMHA199 region between ColEI and trfA was carried out using trfA_pme-F and ColEI_SbfI-R. The fragment was ligated into a modular plasmid pMTL80451 which had it's *tetA* cassette replace with a kanamycin resistant gene.

To obtain CRISPR-Cas9 plasmids, pCas9_Empty_+fdx-term plasmid from the SBRC culture collection carrying Cas9 gene was digested with SbfI and AscI to remove the Cas9 module. The Cas9 module was ligated into pMTL94111 to give pMTL94111-Cas9. pMTL94111-Cas9 was digested and ligated to two different gene strings synthesized by Twist Bioscience. This resulted in pMTL9BR2-Cas9 and pMTL9BR2-Cas9-gRNA_*phaC*. In addition to *S. pyogenes* WT Cas9, PMTL9BR2-Cas9 changes included a fused Cas9 promoter P_{mdh}. pMTL94111-Cas9 had a fused Ferrodoxin promoter P_{fdx}. pMTL9BR2-Cas9-gRNA_*phaC* is identical to pMTL9BR2-Cas9 but also has a gRNA promoter, *phaC* gRNA spacer, a gRNA and a Clostridial ferrodoxin terminator. To make a full Cas9 plasmid with homology arms, HiFi assembly was used. Firstly, primers Phac_LHA_Hifi_F1, Phac_LHA_Hifi_R1, Phac_RHA_Hifi_F2 and Iso12Phac_RHA_R3 were used to amplify *phaC* left and right homology arms from *M. parvus* OBBP genome with Q5 polymerase. pMTL9BR2-Cas9-gRNA_*phaC* was then digested with AsiSI and AscI to provide the backbone for HiFi assembly. Finally, the homology arms were assembled with the backbone in a three-fragment HiFi assembly. This resulted in pMTL9BR2-Cas9_Δ*phaC* which was transformed in *E. coli* DH5α using heat shock according to New England Biolabs (NEB) instructions. After growth of colonies, PCR screening and miniprep, the correct plasmids were heat-shocked into *E. coli* S17 λpir for bi-parental mating with *M. parvus* OBBP.

For plasmid pMTL9BR2-Cas9_*ΔligD* targeting the ATP-dependent DNA ligase *ligD*, spacer gRNA region of *phaC* in pMTL9BR2-Cas9-gRNA_*phaC* was replaced with *ligD* spacer gRNA. Firstly, pMTL9BR2-Cas9-gRNA_*phaC* was digested with PacI and AsiSi to remove

phaC gRNA fused to the *phaC* gRNA spacer. pMTL9BR2-Cas9-gRNA_*phaC* plasmid was then used as a template for PCR amplification with forward primer *ligD*For-sgRNAPrm1 and reverse primer AsiSi_fdxterm-Rev. The forward primer carried a PacI restriction site and *ligD* gRNA spacer as overhang. The reverse primer carried the AsiSi restriction site. The amplicon was then digested and ligated with the digested pMTL9BR2-Cas9-gRNA_*phaC* backbone. After cloning *ligD* gRNA spacer into pMTL9BR2-Cas9-gRNA_*phaC*, pMTL9BR2-Cas9-gRNA_*ligD* was obtained. This plasmid was then digested with AsiSi and AscI to form a HiFi backbone into which 1000 bp left and right *ligD* homology arms are assembled into. This resulted in pMTL9BR2-Cas9_Δ*ligD*.

For subsequent *M. parvus* OBBP plasmids (pMTL9BR2-Cas9-Δ*ligD*_500HA, pMTL9BR2-Cas9_*bcsb*, pMTL9BR2-Cas9_ΔHYP515 and pMTL9BR2-Cas9_Δ*pntA*), pMTL9BR2-Cas9-gRNA_*ligD* was digested with PacI and AscI which was used as a backbone for a four-fragment HiFi including spacer gRNA, left and right homology arms. For genome insertion plasmid pMTL9BR2-Cas9_eYFPKI1, pMTL9BR2-Cas9-gRNA_*ligD* was digested with AsiSi and AscI which was used as backbone for HiFi assembly of *ligD* LHA, eYFP and *ligD* RHA in that order. For insertion plasmid pMTL9BR2-Cas9_eYFPKI2, pMTL9BR2-Cas9-gRNA_*ligD* was digested with AsiSi and AscI which was used as backbone for HiFi assembly of *ligD* LHA up to 244 bp into *ligD*, eYFP and RHA which was from 246 bp to 1246 bp of *ligD*. For guide RNA spacer design of *M. parvus* OBBP, on-Target scoring was calculated using Benchling with *Streptococcus pyogenes* Cas9 specific calculations for on-target scoring [2] and weighted off target scoring [3] calculated against the *M. parvus* OBBP MerPar_1.0 genome. CRISPy-Web (https://crispy.secondarymetabolites.org/#/input) was used for calculating off-target score of *M. capsulatus* Bath [4].

For *M. capsulatus* Bath *mmoX* plasmid (pMTL9BR1-Cas9_*ΔmmoX*), pMTL9BR2-Cas9-gRNA_*ligD* was first digested with PacI and AsiSi to ligate *mmoX* spacer gRNA module. HiFi assembly was then used to add homology arms to the resulting plasmid. Afterwards, P_{mxaf} was PCR amplified and used to replace P_{als} which was the spacer gRNA promoter in pMTL9BR2-Cas9-gRNA_*ligD*. This replacement was necessary because *M. parvus* OBBP promoters showed weak activity in *M. capsulatus* Bath (Figure 1). Because P_{mdh} was fused to Cas9, HiFi was used to clone P_{phac} promoter to Cas9, replacing P_{mdh} and resulting in plasmid pMTL9BR1-Cas9_Δ*mmoX*. To make pMTL9BR1-Cas9_Δ*ligA* plasmid, *ligA* gRNA spacer was PCR amplified and cloned into pMTL9BR1-Cas9_Δ*mmoX*. This was followed by HiFi assembly of *ligA* homology arms to give pMTL9BR1-Cas9_Δ*ligA*. For subsequent *M. capsulatus* Bath CRISPR-Cas9 genome editing plasmids (pMTL9BR1-Cas9_Δ*czcA*, pMTL9BR1-Cas9_ΔMCA_0145 and pMTL9BR1-Cas9_ΔMCA_2158), pMTL9BR1-Cas9_Δ*mmoX* was used as backbone for four-fragment HiFi assembly. Simply, spacer gRNA, left and right homology arms were PCR-amplified with NEB Q5 polymerase. The amplicons were mixed with plasmid pMTL9BR1-Cas9_Δ*mmoX* digested with PacI and AscI in a four-fragment HiFi reaction which was transformed into *E. coli* DH5α and subsequently into *E. coli* S17 λ pir for bi-parental mating into *M. capsulatus* Bath. For gene insertion into *M. capsulatus* Bath, pMTL9BR1-Cas9_ΔMCA_0145 was digested with AsiSi and FseI. PCR amplification of left homology arm, gene insert (eYFP or PHB) and right homology arm was carried out with Q5 polymerase. All four fragments were then assembled using NEB HiFi assembly kit and transformed into *E. coli* DH5α and subsequently into *E. coli* S17 λ pir for bi-parental mating into *M. capsulatus* Bath.

To carry out transposon mutagenesis, a transposon plasmid that works in methanotrophs was first cloned. Simply, primer set R6K_for_PmeI2 and R6K-rev4_SacII was used to amplify R6K

from pUC18R6K-mini-Tn7T-Gm. R6K was the preferred origin of replication due to its suicide replication properties. After digesting the R6K amplicon with PmeI and SacII, it was cloned into pMTLBR-Tn5_Tet-15a (SBRC Nottingham) thus replacing the P15a origin of replication to give pMTLBR-Tn5_Tet. A kanamycin transposon flanked by inner and outer mosaic ends was synthesized by Twist Bioscience and cloned into pMTLBR-Tn5_Tet, replacing the tetracycline transposon with kanamycin transposon to give pMTL90531_Tn5. This plasmid was used for transposon mutagenesis.

After carrying out transposon mutagenesis and obtaining mutant colonies, inverse PCR was used to determine the genomic location of each transposon insertion. It involved genomic DNA extraction; digestion with an enzyme that cuts at the 5’ end of the transposon and several times in the genome of methanotroph; ligation of the digested genomic fragments so they self-ligate; and finally using 3’ – 5’ oriented primers to carry out the PCR. After gDNA extraction, 300 ng of methanotroph gDNA was digested with 1 µL of EcoRI (NEB) in a 50 µL reaction and incubated at 37°C for 2 hrs. The restriction digest enzyme was inactivated at 65°C for 20 minutes. 1 µL of DNA ligase, 2 µL of DNA ligase buffer and 35 µL of NFW were added to 2µL of digest (6ng/µL) and incubated at room temperature (22°C) for 1 hr in a 40 µL ligation mix. DNA ligase was inactivated at 65°C for 10 minutes.

Inverse PCR was carried out using the KOD Hot Start Polymerase. The reaction mix constituted 10 µL of KOD Hot Start Polymerase, 10 mM of forward and reverse primer, 2 µL of ligation mix and NFW to make up a 20 µL ligation mix. The PCR programme was 95°C for 2 minutes to activate the polymerase, 95°C for 20 seconds denaturation, 67°C for annealing and 70°C extension time for 3 minutes 25 seconds. This was run for 40 cycles. A final extension at 70°C for 10 minutes was carried out before pausing at 15°C. The primers KOD_Inv_For and KOD_Inv_Rev were used as forward and reverse primer respectively. After running the PCR products on agarose gel to obtain bands, gel DNA extraction was carried out and DNA was Sanger sequenced with Kan_IG_for primer. Parts of the sequence obtained was cross-referenced with methanotroph genome to determine location of transposon insertion.

Alternative CRISPR Knock Outs Seeds

Having confirmed CRISPR knockouts using a single spacer each in *ligD*, *pntA*, MPA_0518 and *bcsB*, further plasmids were designed and produced with alternative spacers for the same target genes. Six additional *ligD* targeting CRISPR spacers were selected, three were designed against *pntA*, three against MPA_0518 and two against *bcsB*. These were selected for a spread of factors including targeting the template and non-template strand of the gene, 3 different PAMs and a range of lengths along the gene, then prioritizing by on-target score and GC of 40-80%. Listed in Table 1.

Assembly of Alt-KO (Alternative Knock Out) *ligD* Plasmids pMTLBR2-Cas9_Δ*ligD*_Alt1-6 was achieved using HiFi DNA assembly similar to the pMTLBR2-Cas9_Δ*ligD* plasmid. The substituted gRNA’s containing the alternative spacers were produced by PCR combining Asisi_fdxterm-Rev with one of the six AltKO_*ligD*Seed_1-6 primers with the pMTLBR2-Cas9_Δ*ligD* as a template. These were inserted by restriction digestion of the spacer and backbone with PacI and AscI and ligation with the use of Antarctic Phosphatase on the backbone.

The Alt-KO *pntA*, MPA_0518 and *bcsB* plasmids were assembled similarly to that specified for pMTLBR2-Cas9_Δ*pntA*, pMTLBR2-Cas9_ΔMPA_0518 and pMTLBR2-Cas9_Δ*bcsB*. The substituted gRNA’s containing the alternative spacers were produced by PCR combining

AltKO_*pntA*_Seed_1-3 with *pntA*_sgRNA_R, AltKO_MPA_0518_Seed_1-3 with MPA_0518_sgRNA_R and AltKO_*bcsB*_Seed_1-2 with *bcsB*-KO_sgRNA_R all using p9-Cas9 as the template. The backbone was digested with PacI and AscI then the alternative gRNAs, digested backbone, homology arms and gRNA were assembled in a one pot HiFi reaction. The outcome of the Alt-KO plasmid assembly is listed in Table S5. Plasmids were then passed into *M. parvus* OBBP by conjugation then plated on 50µg/ml kanamycin, 25 µg/ml nalidixic acid NMS plates. Grown colonies after 3 weeks under a methane air atmosphere were picked and tested by colony PCR with the appropriate screening primers as described previously.

The knockout success percentage of the 18 original and alternative spacers were plotted against the variables in spacer design (Figure S3 I A-D). Visually a trend appears to show lower knock out success at lower distances from the end of the gene (Figure S3 C). Spearman's Rho (ρ) calculation was carried out as a statistical test of monotonic relationship as it was judged from the figure any relationship was unlikely to be linear. This results in $\rho=0.512$ which passes the critical value $\rho(0.05,18)=0.475$ therefore, there is a significant positive correlation. The non-linearity of this correlation is likely due to the sensitivity of the positioning to the first few bases with less sensitivity to positing within the central section of the gene. The percent rather than absolute distance along the gene was also tested $\rho=0.507$ showing statistical significance. This confirms previous wisdom that spacers should be targeted within the central portion of the gene, and by inspection of the Figure S3 C more specifically more than 200bp from an end to maximise knock out efficiency.

Spearman's Rho test was also performed on the other variables for completeness all of which failed to show correlation as predicted visually. Importantly the lack of trend visually or statistically does not indicate these variables are unimportant or uncorrelated, the values used particularly for GC and on-target score do not fully explore the variable space as great variation was not intended during spacer design.

Conjugation efficiency for each plasmid was taken as colony count of six 10µL aliquots under serial dilution on 50µg/ml kanamycin LB and 50µg/ml kanamycin, 25 µg/ml nalidixic acid NMS plates. This indicated broadly similar efficiencies for all plasmids ($1.00\text{-}4.10 \times 10^{-7}$) except for p9- AltKO_MPA_0518_Seed_2 which had a much higher efficiency (1.71×10^{-3}); as a lower efficiency is expected due to CRISPRs effect on survival, this implies AltKO_MPA_0518_Seed_2 did not cut successfully reflected in its failure to cause any knock outs. Conversely AltKO_MPA_0518_Seed_3 caused no knockouts but did have a CRISPR range conjugation efficiency of 1×10^{-7} suggesting it had been cutting but possibly at an off-target location.

In conclusion the alternative knock outs have been successful in 4 genes of *M. parvus* OBBP using 14 spacers, of which only 2 spacers failed, both against a single gene (MPA_0518) conclusively showing the robustness and effectiveness of this implementation of the CRISPR technique within *M. parvus* OBBP.

Table S1. Strains used in this study.

Strain	Description	Reference
***E.coli* DH5α**	fhuA2 Δ(argF-lacZ)U169 phoA glnV44 Φ80 Δ(lacZ)M15 gyrA96 recA1 relA1 endA1 thi-1 hsdR17	[5]
***E.coli* S17-1 λpir**	*TpR SmR recA, thi, pro, hsdR-M+RP4: 2-Tc:Mu: Km Tn7 λpir.*	[6]
***E.coli* XL-1 Blue**	*recA1 endA1 gyrA96 thi-1 hsdR17 supE44 relA1 lac* [F′ *proAB lacIqZΔM15 Tn10* (TetR)	[7]
***M. parvus* (OBBP)**	Type II *Methylocystis* species (Wild Type)	NCIMB 11129
***M. parvus* (BRCS2)**	Type II *Methylocystis* species isolated from peat bog (Wild Type)	NCIMB 15262
***M. capsulatus* Bath**	Type I *Methylococcus* specie (Wild Type)	Kindly gifted by Dr Andrew Crombie (University of East Anglia)

Table S2. List of promoters and origin.

Promoter	**Full name of genes promoters originated from**	**Species promoters originated from**
**Phik**	Histidine Kinase	*M. parvus* OBBP
**Pmdh**	Methanol dehydrogenase	*M. parvus* OBBP
**Phps**	3-Hexulose-6-phosphate synthase	*M. capsulatus* Bath
**Pals**	acetolactate synthase	*M. capsulatus* Bath
**Pphac**	Polyhdroxyalkanoate promoter	*C. necator* H16
**P3**	Synthetic promoters	Synthetic promoter [8]
**Pmxaf**	Methanol dehydrogenase	*M. capsulatus* Bath
**Pnpr01**	Synthetic promoters	Synthetic promoter [8]
**P13**	Synthetic promoters	Synthetic promoter [8]

Table S3. List of primers used for designing plasmids and screening gene deletions.

Name	Sequence (5' - 3')	Additional Information
phaC gene deletion		
***phaC*_LHA_Hifi_F1**	TTCTTATTTTTATGCGATCGATCGTCATCGCAGGCA	LHA
***phaC*_LHA_Hifi_R1**	CAACAATTCCGGCTGAGGGGCCAGAATCCTGACCGG	
***phaC*_RHA_Hifi_F2**	CCGGTCAGGATTCTGGCCCCTCAGCCGGAATTGTTG	RHA
**Iso12Phac_RHA_R3**	GCCGGCCCAGTCGGCGCAGGAAGCGGTGATACATC	
***phaC* _LHAF_OHA**	ACTTTGGGCCAAGACCTTC	Screening
***phaC*_RHA_OHA2**	GACGAGACGCCTGATTTCG	
ligD gene deletion and addition		
***ligD*_LHA-FWD**	AGGCTTCTTATTTTTATGCGATTTTCTTTTTGCGTATCTCCGGC	LHA
***ligD*_LHA-REV**	GCCGCGCAACGCCTCGCAATGTGGATCAAGCCCGGCGGCGCGTT	
***ligD*_RHA-FWD**	AACGCGCCGCCGGGCTTGATCCACATTGCGAGGCGTTGCGCGGC	RHA
***ligD*_RHA_Rev2**	GCCGGCCCAGTCGGCGCGGGCGCAAGGAGCTGGACCTCGC	
**ligD_KO_500bp_1F**	GCTTCTTATTTTTATGCGATGGTCCAAGCGAAAGGTTG	LHA
**ligD_KO_500bp_1R**	CGCGCAACGCCTCGCAATGTGGATCAAGCCCGGCG	
**ligD_KO_500bp_2F**	CGCGCCGCCGGGCTTGATCCACATTGCGAGGCGTTGC	RHA
**ligD_KO_500bp_2R**	TGGCCGGCCCAGTCGGCGCGATCAGCGAATTGATCGGCG	
**2*ligD*-LHA_Pro-F**	GCTTCTTATTTTTATGCGATTTTCTTTTTGCGTATCTCCGG	LHA
**2*ligD*-LHA_Pro-R**	CCCTCAGCCTTAAGAAACGCGGATCAAGCCCGGCG	
**2*ligD*-RHA_Pro-F**	AGCCTGAATGGCGAATGGCGACATTGCGAGGCGTTGCG	RHA
**2*ligD*-RHA_Pro-R**	GCCGGCCCAGTCGGCGCGACCTCGCGCTCGCCC	
**2*ligD*_ProEYFP-F**	GCGCCGCCGGGCTTGATCCGCGTTTCTTAAGGCTGAGG	eYFP knock in (replacement)
**2*ligD*_ProEYFP-R**	GCGCAACGCCTCGCAATGTCGCCATTCGCCATTCAG	
**2*ligD*_EYFPonly-F**	GCGCCGCCGGGCTTGATCCATGGTGAGCAAGGGCG	
**ligD_2_P3EYKI_1F**	CCCTCAGCCTTAAGAAACGCCGGACCGCAAGGCG	LHA
**ligD_2_P3EYKI_1R**	CCCTCAGCCTTAAGAAACGCCGGACCGCAAGGCG	
**ligD_2_P3EYKI_2F**	GACCGCCGCCTTGCGGTCCGGCGTTTCTTAAGGCTGAGGG	eYFP knock in
**ligD_2_P3EYKI_2R**	CGAGAGGATGGTCTTCCGTGCGCCATTCGCCATTCAGG	

**ligD_2_P3EYKI_3F**	AGCCTGAATGGCGAATGGCGCACGGAAGACCATCCTCTC	RHA
**ligD_2_P3EYKI_3R**	TGGCCGGCCCAGTCGGCGCGCACATCCTCTCGAAAGTCG	
pntA gene deletion		
***pntA*_sgRNA_F**	AAGAGGGGCCGAAGCTTAATGGACGCGGCGACGACGCTCGGTTTTAGAGCTAGAAATAGCAAGTTAAAATAAGGC	Guide RNA spacer amplification
***pntA*_sgRNA_R**	TCGACGCGGTCCTTCTTCTCGGCGCGCCGCGATCG	
***pntA*_LHA_F**	TTATGCGATCGCGGCGCGCCGAGAAGAAGGACCGCGTCGA	LHA
***pntA*_LHA_R**	TTTGAGGGATGCTATGCGGCGACGAACTCCGGTTTCGAGA	
***pntA*_RHA_F**	TCTCGAAACCGGAGTTCGTCGCCGCATAGCATCCCTCAAAC	RHA
***pntA*_RHA_R**	TGGCCGGCCCAGTCGGCGCGTCGGTCGAGACGAAAAGCAG	
***pntA*-KO_OHA_F**	GAAGCTCGGCTATCTCTCGAG	Gene knockout Screening
***pntA*-KO_OHA_R**	TGGCGGGATAATAATTGTCG	
Hypothetical protein 518 (MCA_0518) gene deletion		
**MPA_0518_sgRNA_F**	AAGAGGGGCCGAAGCTTAATGGAGCCGGAATCGCGTCGCGGTTTTAGAGCTAGAAATAGCAAGTTAAAATAAGGC	Guide RNA spacer amplification
**MPA_0518_sgRNA_R**	CGACCACGACCTATCGCTACGGCGCGCCGCGATC	
**MPA_0518_LHA_F**	GATCGCGGCGCGCCGTAGCGATAGGTCGTGGTCG	LHA
**MPA_0518_LHA_R**	GCGGCGACTCATTCAATCCTGCGGTCTCCTATGGCG	
**MPA_0518_RHA_F**	CAAGCGCCATAGGAGACCGCAGGATTGAATGAGTCGCCGCAC	RHA
**MPA_0518_RHA_R**	TGGCCGGCCCAGTCGGCGCGCTTCGCCGCGTCCGGAG	
**HYPROT_seq-F**	CAGATAGAGCCCGTTCATCAG	Gene knockout Screening
**HYPROT_seq-R**	TGGTAGAGATCGACATAATCGG	
bcsB gene deletion		
***bcsB*_sgRNA_F**	AAGAGGGGCCGAAGCTTAATCACGCCGGTGAATACGACGCGTTTTAGAGCTAGAAATAGCAAGTTAAAATAAGGC	Guide RNA spacer amplification
***bcsB*_sgRNA_R**	TGAAGGCCACCGCTTCGTCGGGCGCGCCGCGATCG	
***bcsB*-KO_LHA_F**	TTATGCGATCGCGGCGCGCCCGACGAAGCGGTGGCCTTC	LHA
***bcsB*-KO_LHA_R**	GGCGACGCGGAGCGCGAAGCTCATGCCGATTTGCGCGC	
***bcsB*-KO_RHA_F**	CGGCGCGCAAATCGGCATGAGCTTCGCGCTCCGCG	RHA
***bcsB*-KO_RHA_R**	TGGCCGGCCCAGTCGGCGCGTCGACGCCGTTCGTCACG	
***bcsB*_seq-F**	CGACCGTGAGAAGCTCCAGG	Gene knockout Screening

***bcsB*_seq_R**	GATCGGGATGATCAGCGTCAC	
MCA_0145 gene deletion		
**MCA_0145-Seed_F**	TAGTAGTGGAGGAGATTAATTAAGAATCTGTCTACGCTCAACGGTTTTAGAGCTAGAAATAGCAAGT	Guide RNA spacer amplification
**MCA_0145-Seed_R**	GCCGCCAGTAAACACCGATGGCGATCGCATAAAAATAAGAAGCCTGCAAAT	
**MCA_0145-LHA_F**	TGCAGGCTTCTTATTTTTATCATCGGTGTTTACTGGCG	LHA
**MCA_0145-LHA_R**	ATCAGCGACTCCTGTCAGACTTACGGAAATGTCCATATTGGA	
**MCA_0145-RHA_F**	CAATATGGACATTTCCGTAAGTCTGACAGGAGTCGC	RHA
**MCA_0145-RHA_R**	CCAGTAGCTGACATGGCCGGCCTATCCTCCATGGAGTGAACA	
**MCA_0145_KO_Screen_F**	GGGTGTTCGAAAATTATCTCGACG	Gene knockout Screening
**MCA_0145_KO_Screen_R**	GAAGCGCTGCTTAATGCTCC	
czcA gene deletion		
***czcA*-Seed_F**	TAGTAGTGGAGGAGATTAATCGTCAAGCAATACCAGGTGTGTTTTAGAGCTAGAAATAGCAAGT	Guide RNA spacer amplification
***czcA*-Seed_R**	GTTCACCTTCGGACACCACGGCGATCGCATAAAAATAAGAAGCCTGCAAATG	
***czcA*-LHA_F**	TGCAGGCTTCTTATTTTTATCGTGGTGTCCGAAGGTG	LHA
***czcA*-LHA_R**	CCTGATTTTATGGTTCGCGCCGATTACTGCTCCAGCTCC	
***czcA*-RHA_F**	GGGAGCTGGAGCAGTAATCGGCGCGAACCATAAAATCAGG	RHA
***czcA*-RHA_R**	CCAGTAGCTGACATGGCCGGCCCAATCCCGGCATGGCC	
***czcA*_KO_Screen_F**	GGTTTTTCTGTGGGCCTGTC	Gene knockout Screening
***czcA*_1298_KO_Screen_R**	ATGCGACCACTGGATACTGA	
MCA_2158 gene deletion		
**MCA_2158-Seed_F**	TAGTAGTGGAGGAGATTAATGCAGCGGACCGAATTCTTTCGTTTTAGAGCTAGAAATAGCAAGT	Guide RNA spacer amplification
**MCA_2158-Seed_R**	AGAAAGCCGACGAAGCCCAGGCGATCGCATAAAAATAAGAAGCCTGCAAATG	
**MCA_2158-LHA_F**	TGCAGGCTTCTTATTTTTATCTGGGCTTCGTCGGCTTTCT	LHA
**MCA_2158-LHA_R**	GTCGCCAGGGAGATATTTCGCCGGGAAAAGCGGAGAC	
**MCA_2158-RHA_F**	CCCGTCTCCGCTTTTCCCGGCGAAATATCTCCCTGGCG	RHA
**MCA_2158-RHA_R**	CCAGTAGCTGACATGGCCGGCCGCGGACCCGATAAATGC	
**MCA2518_KO_Screen_F**	TTCGGCGTACACAACGAGAT	Gene knockout Screening
**MCA2518_KO_Screen_R**	CAAATTTGGGGGCTGCCTG	
eYFP gene insertion M.c Bath		
**Mc145-P3KI_1F**	GCGCTGTCAATCTAGCTAGAGAGGTTCAGGCG	LHA

**Mc145-P3KI_1R**	AGCCTTAAGAAACGCTTACGGAAATGTCCATATTGG	
**Mc145-P3KI_2F**	TGGACATTTCCGTAAGCGTTTCTTAAGGCTGA	P3+eYFP
**Mc145-P3KI_2R**	CGACTCCTGTCAGACCGCCATTCGCCATTC	
**Mc145-P3KI_3F**	GAATGGCGAATGGCGGTCTGACAGGAGTCGC	RHA
**Mc145-P3KI_3R**	TCAGTTCCGGCGTTTAAACTCATTTCGAACCCC	
PHB gene insertion M. c Bath		
**Mc145-PhaC_1F**	GCGCTGTCAATCTAGCTAGAGAGGTTCAGGCG	LHA
**Mc145-PhaC_1R**	ATCCGCCTCGGCACTTTACGGAAATGTCCATATTGG	
**Mc145-PhaC_2F**	TGGACATTTCCGTAAAGTGCCGAGGCGGAT	phaCAB
**Mc145-PhaC_2R**	CGACTCCTGTCAGACTCAGCCCATATGCAGG	
**Mc145-PhaC_3F**	CTGCATATGGGCTGAGTCTGACAGGAGTCGC	RHA
**Mc145-PhaC_3R**	TCAGTTCCGGCGTTTTATCCTCCATGGAGTGAAC	
Additional gene deletions		
**AltKO_*ligD*_Seed_1**	TAAAGAGGGGCCGAAGCTTAATTAA CGAGAGGATGGTCTTCCGTG GTTTTAGAGCTAGAAATAGCAAGTT	Primers for designing alternative guide RNA spacers of respective genes
**AltKO_*ligD*_Seed_2**	TAAAGAGGGGCCGAAGCTTAATTAA ATGGTCGCGAATTTCCCCCG GTTTTAGAGCTAGAAATAGCAAGTT	
**AltKO_*ligD*_Seed_3**	TAAAGAGGGGCCGAAGCTTAATTAA CATCACCCATGCAAGCCGGG GTTTTAGAGCTAGAAATAGCAAGTT	Primers for designing alternative guide RNA spacers of respective genes<br><br>Plasmid design
**AltKO_*ligD*_Seed_4**	TAAAGAGGGGCCGAAGCTTAATTAA GCGCCATATAAAGTTCGTCG GTTTTAGAGCTAGAAATAGCAAGTT	
**AltKO_*ligD*_Seed_5**	TAAAGAGGGGCCGAAGCTTAATTAA TTTCAGCTCGAAGAGCCAA TGTTTTAGAGCTAGAAATAGCAAGTT	
**AltKO_*ligD*_Seed_6**	TAAAGAGGGGCCGAAGCTTAATTAA GATCAAGGGCGACTTTCGAG GTTTTAGAGCTAGAAATAGCAAGTT	
**AltKO_*pntA*_Seed_1**	AAGAGGGGCCGAAGCTTAAT TTTGAAACGTTCGTCCGTAA GTTTTAGAGCTAGAAATAGCAAGTTAAAATAAGGC	
**AltKO_*pntA*_Seed_2**	AAGAGGGGCCGAAGCTTAAT TCATCTCGAAGGAGACGAAG GTTTTAGAGCTAGAAATAGCAAGTTAAAATAAGGC	
**AltKO_*pntA*_Seed_3**	AAGAGGGGCCGAAGCTTAAT CTTCGCGTAGAGGCTCGAAG GTTTTAGAGCTAGAAATAGCAAGTTAAAATAAGGC	
**AltKO_MPA_0518_Seed_1**	AAGAGGGGCCGAAGCTTAAT TTCGACACCGAGACGCCGCG GTTTTAGAGCTAGAAATAGCAAGTTAAAATAAGGC	
**AltKO_MPA_0518_Seed_2**	AAGAGGGGCCGAAGCTTAAT AAGCGGTAGAGCGTGCCCCG GTTTTAGAGCTAGAAATAGCAAGTTAAAATAAGGC	
**AltKO_MPA_0518_Seed_3**	AAGAGGGGCCGAAGCTTAAT AAGGCCGACCAGAACCACGA GTTTTAGAGCTAGAAATAGCAAGTTAAAATAAGGC	
**AltKO_*bcsB*_Seed_1**	AAGAGGGGCCGAAGCTTAAT TCGTCAAGAATGTCGGCAGG GTTTTAGAGCTAGAAATAGCAAGTTAAAATAAGGC	
**AltKO_*bcsB*_Seed_2**	AAGAGGGGCCGAAGCTTAAT GTATGTGAACGAGCGCACGG GTTTTAGAGCTAGAAATAGCAAGTTAAAATAAGGC	
Transposon primers		
**R6K_for_PmeI2**	TACAAGCAGTTTAAACGCAGTTCAACCTGTTGATAGTAC	

**R6K-rev4_SacII**	TAGATACCGCGGTTGTCAGCCGTTAAGTG	
**Kan_IV_For_KOD**	GATTCGCAGCGCATCGCCTTCTATCGCCTTCTTGAC	Inverse PCR
**Kan_IV_Rev_KOD**	GCCATCCAGTTTACTTTGCAGGGCTTCCCAACCTTAC	
**Kan_IG_for**	GAACAAGATGGATTGCACGC	Sequencing

Table S4. List of plasmids used in this study.

Plasmids	Description	Reference
**pMTL94111**	oriV, oriT, trfA, ColE1, kan^R	[9]
**pMTL94115**	oriV, oriT, trfA, ColE1, kan^R, eYFP	[9]
**pMTL94115_P*hik***	oriV, oriT, trfA, ColE1, kan^R, eYFP, P*phik*	This study
**pMTL94115_P*phaC***	oriV, oriT, trfA, ColE1, kan^R, eYFP, P*phaC*	This study
**pMTL94115_P*mdh***	oriV, oriT, trfA, ColE1, kan^R, eYFP, P*mdh*	This study
**pMTL94115_P*als***	oriV, oriT, trfA, ColE1, kan^R, eYFP, P*als*	This study
**pMTL94115_P*hps***	oriV, oriT, trfA, ColE1, kan^R, eYFP, P*hps*	This study
**pMTL94115_Pnpr01**	oriV, oriT, trfA, ColE1, kan^R, eYFP, Pnpr01	This study
**pMTL94115_P13**	oriV, oriT, trfA, ColE1, kan^R, eYFP, P13	This study
**pMTL94115_P3**	oriV, oriT, trfA, ColE1, kan^R, eYFP, P3	This study
**pMTL94115_P*mxaF***	oriV, oriT, trfA, ColE1, kan^R, eYFP, P*mxaF*	This study
**pCas9_Empty_+fdx-term**	ColE1, traJ, Cm^R, Cas9	[10]
**pMTL9BR2-Cas9**	oriV, oriT, trfA, ColE1, kan^R, Cas9 , P_{mdh}	This study
**pMTL9BR2-Cas9pals-gRNA_*phaC***	oriV, oriT, trfA, ColE1, kan^R,Cas9, $P_{als,}$ P_{mdh}, *phaC* sgRNA, *t*fdx	This study
**pMTL9BR2-Cas9Pals-Δ*phaC***	oriV, oriT, trfA, ColE1, kan^R,Cas9, $P_{als,}$ P_{mdh}, *phaC* sgRNA, *t*fdx, *phaC* LHA, *phaC* RHA	This study
**pMTL9BR2-Cas9-gRNA_*phaC***	oriV, oriT, trfA, ColE1, kan^R,Cas9, P_{mdh}, P_{als}, *phaC* sgRNA, *t*fdx	This study
**pMTL9BR2-Cas9-Δ*phaC***	oriV, oriT, trfA, ColE1, kan^R,Cas9, P_{mdh}, P_{als}, *phaC* sgRNA, *t*fdx, *phaC* LHA, *phaC* RHA	This study
**pMTL9BR2-Cas9-gRNA_*ligD***	oriV, oriT, trfA, ColE1, kan^R,Cas9, P_{mdh}, P_{als}, *ligD* sgRNA, *t*fdx	This study
**pMTL9BR2-Cas9_Δ*ligD***	oriV, oriT, trfA, ColE1, kan^R,Cas9, P_{mdh}, P_{als}, *ligD* sgRNA, *t*fdx, *ligD* LHA, *ligD* RHA	This study
**pMTL9BR2-Cas9_Δ*copD***	oriV, oriT, trfA, ColE1, kan^R,Cas9, P_{mdh}, P_{als}, *copD* sgRNA, *t*fdx, *copD* LHA, *copD* RHA	This study
**pMTL9BR2-Cas9_Δ*glg***	oriV, oriT, trfA, ColE1, kan^R,Cas9, P_{mdh}, P_{als}, *glg* sgRNA, *t*fdx, *glg* LHA, *glg* RHA	This study
**pMTL9BR2-Cas9_Δ*ligA***	oriV, oriT, trfA, ColE1, kan^R,Cas9, P_{mdh}, P_{als}, *ligA* sgRNA, *t*fdx, *ligA* LHA, *ligA* RHA	This study
**pMTL9BR2-Cas9-Δ*ligD*_500HA**	oriV, oriT, trfA, ColE1, kan^R,Cas9, P_{mdh}, P_{als}, *ligD* sgRNA, *t*fdx, *ligD* LHA, *ligD* RHA (500bp)	This study
**pMTL9BR2-Cas9_Δ*pntA***	oriV, oriT, trfA, ColE1, kan^R,Cas9, P_{mdh}, P_{als}, *pntA* sgRNA, *t*fdx, *pntA* LHA, *pntA* RHA	This study
**pMTL9BR2-Cas9_ΔMPA_0518**	oriV, oriT, trfA, ColE1, kan^R,Cas9, P_{mdh}, P_{als}, MPA_0518 sgRNA, *t*fdx, MPA_0518 LHA, MPA_0518 RHA	This study
**pMTL9BR2-Cas9_Δ*bcsB***	oriV, oriT, trfA, ColE1, kan^R,Cas9, P_{mdh}, P_{als}, *bcsB* sgRNA, *t*fdx, *bcsB* LHA, *bcsB* RHA	This study
**pMTL9BR2-Cas9_eYFPKI1**	oriV, oriT, trfA, ColE1, kan^R,Cas9, P_{mdh}, P_{als}, *ligD* sgRNA, *t*fdx, *ligD* LHA, eYFP, *ligD* RHA	This study
**pMTL9BR2-Cas9_eYFPKI2**	oriV, oriT, trfA, ColE1, kan^R,Cas9, P_{mdh}, P_{als}, *ligD* sgRNA, *t*fdx, *ligD* LHA, eYFP, *ligD* RHA	This study
**pMTL9BR1-Cas9_Δ*mmoX***	oriV, oriT, trfA, ColE1, kan^R,Cas9, P_{phac}, P_{mxaf}, *mmoX* sgRNA, *t*fdx, *mmoX* LHA, *mmoX* RHA	This study
**pMTL9BR1-Cas9_ΔMCA_0145**	oriV, oriT, trfA, ColE1, kan^R,Cas9, P_{phac}, P_{mxaf}, MCA_0145 sgRNA, *t*fdx, MCA_0145 LHA, MCA_0145 RHA	This study
**pMTL9BR1-Cas9_ Δ*czcA***	oriV, oriT, trfA, ColE1, kan^R,Cas9, P_{phac}, P_{mxaf}, MCA_1298 sgRNA, *t*fdx, MCA_1298 LHA, MCA_1298 RHA	This study
**pMTL9BR1-Cas9_ΔMCA_2158**	oriV, oriT, trfA, ColE1, kan^R,Cas9, P_{phac}, P_{mxaf}, MCA_2158 sgRNA, *t*fdx, MCA_2158 LHA, MCA_2158 RHA	This study
**pMTL9BR1-Cas9_Δ*McligA***	oriV, oriT, trfA, ColE1, kan^R,Cas9, P_{phac}, P_{mxaf}, *McligA* sgRNA, *t*fdx, *McligA* LHA, *McligA* RHA	This study
**pMTL9BR1-Cas9_Δ*Mc*eYFPKI1**	oriV, oriT, trfA, ColE1, kan^R,Cas9, P_{phac}, P_{mxaf}, MCA_0145 sgRNA, *t*fdx,MCA_0145 LHA, eYFP, MCA_0145 RHA	This study
**pUC18R6K-mini-Tn7T-Gm**	R6K, traj, Gm^R, Tn7	[11]
**pMTLBR-Tn5_Tet-P15a**	P15a, traj, tetr^R, Tn5, TnpA	This study

**pMTLBR-Tn5_Tet**	R6K, traj, $tetr^R$, Tn5, TnpA	This study
**pMTL90531_Tn5**	R6K, traj, kan^R, Tn5, TnpA	This study
**pMTLBR2-Cas9_Δ*ligD*_Alt1**	oriV, oriT, trfA, ColE1, kanR,Cas9, P_{mdh}, P_{als}, *ligD* sgRNA Alt1, tfdx, *ligD* LHA, *ligD* RHA	This study
**pMTL9BR2-Cas9_Δ*ligD*_Alt2**	oriV, oriT, trfA, ColE1, kanR,Cas9, P_{mdh}, P_{als}, *ligD* sgRNA Alt2, tfdx, *ligD* LHA, *ligD* RHA	This study
**pMTL9BR2-Cas9_Δ*ligD*_Alt3**	oriV, oriT, trfA, ColE1, kanR,Cas9, P_{mdh}, P_{als}, *ligD* sgRNA Alt3, tfdx, *ligD* LHA, *ligD* RHA	This study
**pMTL9BR2-Cas9_Δ*ligD*_Alt4**	oriV, oriT, trfA, ColE1, kanR,Cas9, P_{mdh}, P_{als}, *ligD* sgRNA Alt4, tfdx, *ligD* LHA, *ligD* RHA	This study
**pMTL9BR2-Cas9_Δ*ligD*_Alt5**	oriV, oriT, trfA, ColE1, kanR,Cas9, P_{mdh}, P_{als}, *ligD* sgRNA Alt5, tfdx, *ligD* LHA, *ligD* RHA	This study
**pMTL9BR2-Cas9_Δ*ligD*_Alt6**	oriV, oriT, trfA, ColE1, kanR,Cas9, P_{mdh}, P_{als}, *ligD* sgRNA Alt6, tfdx, *ligD* LHA, *ligD* RHA	This study
**pMTL9BR2-Cas9_Δ*pntA*_Alt1**	oriV, oriT, trfA, ColE1, kanR,Cas9, P_{mdh}, P_{als}, *pntA* sgRNA Alt1, tfdx, *pntA* LHA, *pntA* RHA	This study
**pMTL9BR2-Cas9_Δ*pntA*_Alt2**	oriV, oriT, trfA, ColE1, kanR,Cas9, P_{mdh}, P_{als}, *pntA* sgRNA Alt2, tfdx, *pntA* LHA, *pntA* RHA	This study
**pMTL9BR2-Cas9_Δ*pntA*_Alt3**	oriV, oriT, trfA, ColE1, kanR,Cas9, P_{mdh}, P_{als}, *pntA* sgRNA Alt3, tfdx, *pntA* LHA, *pntA* RHA	This study
**pMTL9BR2-Cas9_ΔMPA_0518_Alt1**	oriV, oriT, trfA, ColE1, kanR,Cas9, P_{mdh}, P_{als}, MPA_0518 sgRNA Alt1, tfdx, MPA_0518 LHA, MPA_0518 RHA	This study
**pMTL9BR2-Cas9_ΔMPA_0518_Alt2**	oriV, oriT, trfA, ColE1, kanR,Cas9, P_{mdh}, P_{als}, MPA_0518 sgRNA Alt2, tfdx, MPA_0518 LHA, MPA_0518 RHA	This study
**pMTL9BR2-Cas9_ΔMPA_0518_Alt3**	oriV, oriT, trfA, ColE1, kanR,Cas9, P_{mdh}, P_{als}, MPA_0518 sgRNA Alt3, tfdx, MPA_0518 LHA, MPA_0518 RHA	This study
**pMTL9BR2-Cas9_Δ*bcsB*_Alt1**	oriV, oriT, trfA, ColE1, kanR,Cas9, P_{mdh}, P_{als}, *bcsB* sgRNA Alt1, tfdx, *bcsB* LHA, *bcsB* RHA	This study
**pMTL9BR2-Cas9_Δ*bcsB*_Alt2**	oriV, oriT, trfA, ColE1, kanR,Cas9, P_{mdh}, P_{als}, *bcsB* sgRNA Alt2, tfdx, *bcsB* LHA, *bcsB* RHA	This study

Transposon mutagenesis

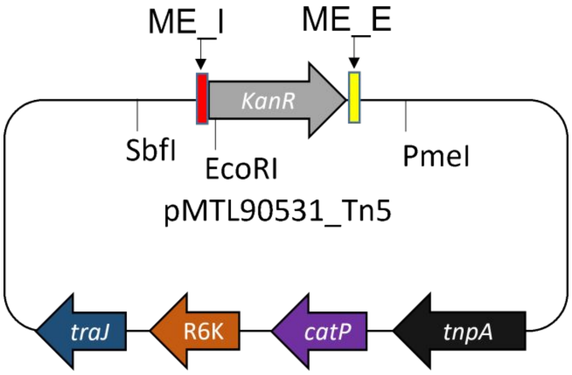

Figure S1. pMTL90531_Tn5 plasmid: This was used for transposon mutagenesis in *M. parvus* BRCS2. ME_I represent the Internal Mosaic End and ME_E represent the External Mosaic End both of which flank the kanamycin resistant gene which plays the role of the transposable element in the plasmid.

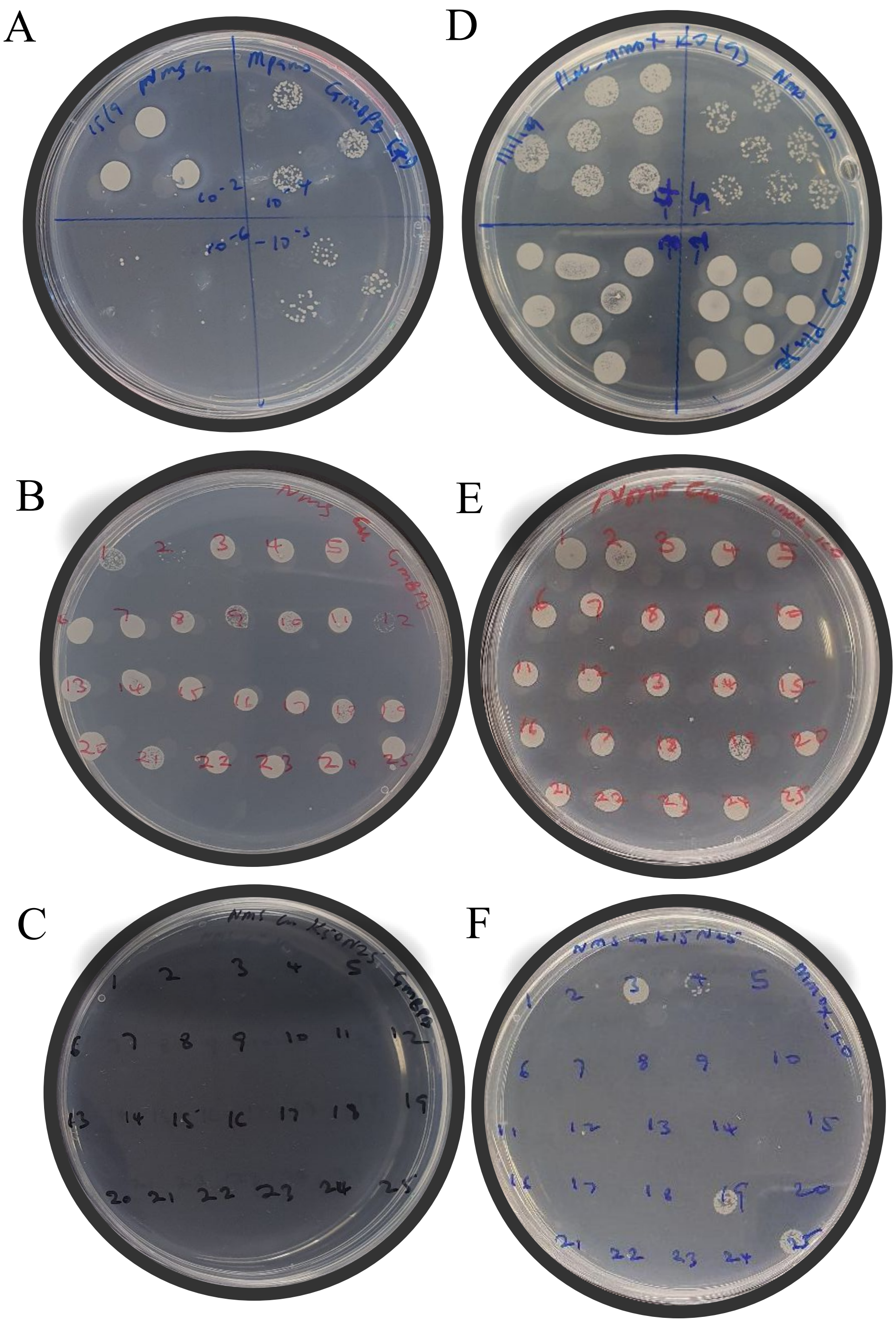

Figure S2. Illustration of plasmid curing on NMS agar plates (A) Dilutions of *M. parvus* OBBP *bcsB* gene deletion mutant grown in NMS media (B) Colonies from (A) grown in NMS media and spotted (5 µL) on NMS bacto agar plate (C) Colonies from (A) grown in NMS media and spotted (5 µL) on NMS kanamycin 50 µg/mL and Nalidixic acid 25 µg/mL bacto agar plate (D) Dilutions of *M. capsulatus* Bath *mmoX* gene deletion mutant grown in NMS media (E) Colonies from (D) grown in NMS media and spotted (5 µL) on NMS bacto agar plate (F) Colonies from (D) grown in NMS media and spotted (5 µL) on NMS kanamycin 15 µg/mL and Nalidixic acid 25 µg/mL bacto agar plate.

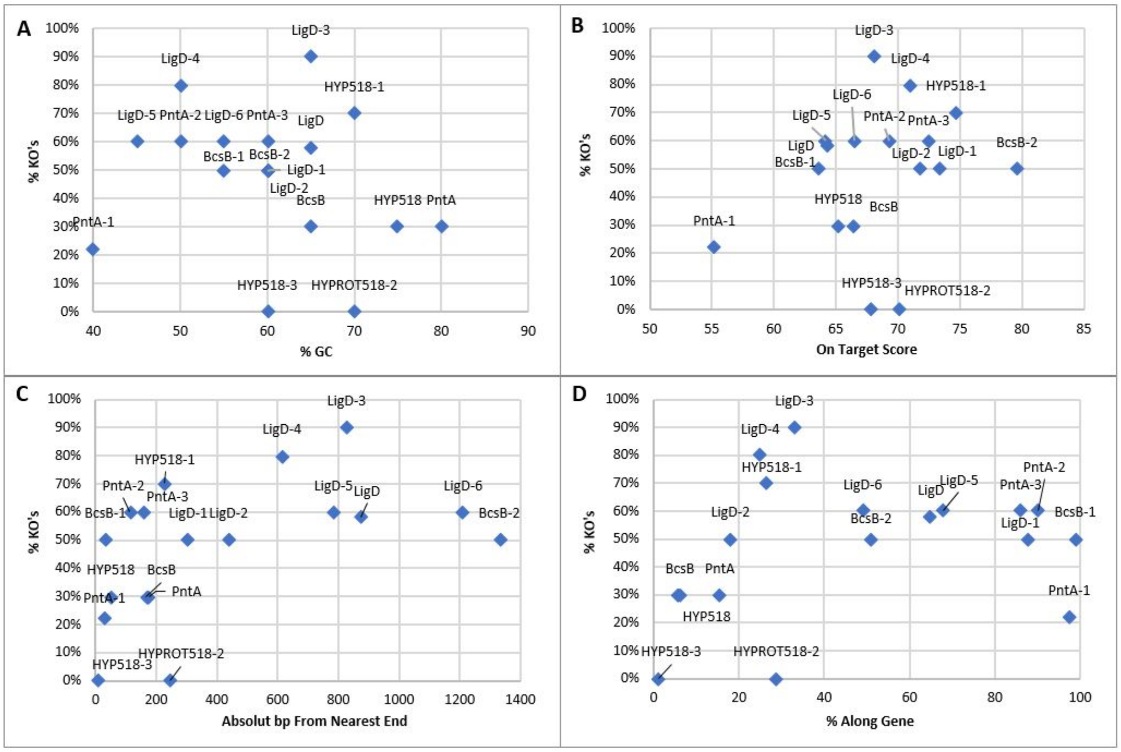

Figure S3. A-D: Graphs of the knock-out success percentages of the 18 tested CRISPR Knock out spacer designs including data across 4 different genes. Plotted against 4 potential influences on spacer effectiveness A) GC Percentage B) On Target Score C) Absolute Base pair distance from the nearest gene end D) Percent distance along the gene, rescaling the data in C to account for varied gene lengths.

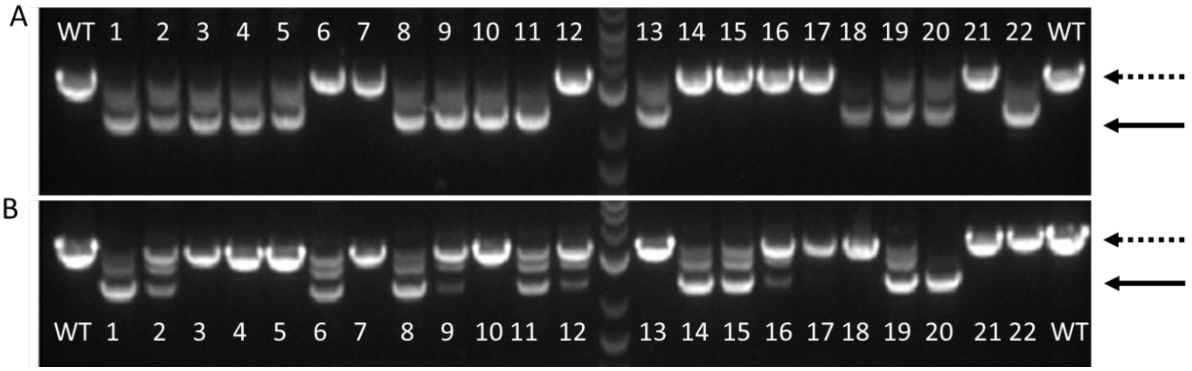

Figure S4. Comparison of MPA_0518 gene knock out in *M. parvus* OBBP Wild Type strain (A) and *M. parvus* OBBP *ΔligD* strain (B) to determine the effect of *ligD* gene deletion on gene knockout efficiency. Wild Type (WT) PCR screens at the beginning and end of gel using *M. parvus* OBBP wild type genomic DNA. NEB 1 kb Plus DNA ladder was used. Dotted lines represent size of WT PCR amplicon while solid lines represent mutants. Lanes 1-22 represent the 22 colonies screened.

References

1- Ali H, Murrell JC. Development and validation of promoter-probe vectors for the study of methane monooxygenase gene expression in *Methylococcus capsulatus* Bath. Microbiology. 2009 Mar 1;155(3):761-71.
2- Doench JG, Fusi N, Sullender M, Hegde M, Vaimberg EW, Donovan KF, Smith I, Tothova Z, Wilen C, Orchard R, Virgin HW. Optimized sgRNA design to maximize activity and minimize off-target effects of CRISPR-Cas9. Nature biotechnology. 2016 Feb;34(2):184-91.
3- Hsu PD, Scott DA, Weinstein JA, Ran FA, Konermann S, Agarwala V, Li Y, Fine EJ, Wu X, Shalem O, Cradick TJ. DNA targeting specificity of RNA-guided Cas9 nucleases. Nature biotechnology. 2013 Sep;31(9):827-32.
4- Blin K, Pedersen LE, Weber T, Lee SY. CRISPy-web: an online resource to design sgRNAs for CRISPR applications. Synthetic and Systems Biotechnology. 2016 Jun 1;1 (2):118-21.
5- NEB, NEB® 5-alpha Competent *E. coli* (High Efficiency). 2018. https://international.neb.com/products/c2987-neb-5-alpha-competent-e-coli-high-efficiency#Product%20Information. Accessed 13 January 2018.
6- Biomedal, *E.coli* S17-1 lambda pir. 2018.
7- Agilent, E.coli XL-1 Blue. 2018. https://www.agilent.com/store/productDetail.jsp?catalogId=200249&catId=SubCat3ECS_227986. Accessed 22 April 2018
8- Alagesan S, Hanko EK, Malys N, Ehsaan M, Winzer K, Minton NP. Functional genetic elements for controlling gene expression in *Cupriavidus necator* H16. Applied and environmental microbiology. 2018 Oct 1;84(19):e00878-18.
9- Plasmid Vectors, Vector systems created at the BBSRC/EPSRC Synthetic Biology Research Centre (SBRC), Nottingham. 2021. https://plasmidvectors.com/clostridium/
10- Cañadas IC, Groothuis D, Zygouropoulou M, Rodrigues R, Minton NP. RiboCas: a universal CRISPR-based editing tool for *Clostridium*. ACS synthetic biology. 2019 Jun 7;8(6):1379-90.
11- Choi KH, Schweizer HP. mini-Tn7 insertion in bacteria with single attTn7 sites: example *Pseudomonas aeruginosa*. Nature protocols. 2006 Jun;1(1):153-61.
